# A Phytosociological Study on Andean Rainforests of Peru, and a Comparison with the Surrounding Countries

**DOI:** 10.3390/plants9121654

**Published:** 2020-11-26

**Authors:** Antonio Galán-de-Mera, José Campos-de-la-Cruz, Eliana Linares-Perea, Juan Montoya-Quino, Iván Torres-Marquina, José Alfredo Vicente-Orellana

**Affiliations:** 1 Laboratory of Botany, San Pablo-CEU University, CEU Universities, Boadilla del Monte, 28660 Madrid, Spain; avicore@ceu.es; 2 Natural History Museum, Major National University of San Marcos, 15046 Lima, Peru; jocamde@gmail.com; 3 Phytogeographical Studies of Peru Foundation, Paucarpata, 04007 Arequipa, Peru; elialinper@gmail.com; 4 CPUN Herbarium, National University of Cajamarca, 06003 Cajamarca, Peru; juanfmq9@gmail.com; 5 Baños del Inca, 06004 Cajamarca, Peru; itorresm@hotmail.com

**Keywords:** rainforests, phytosociology, syntaxonomy, Peru, South America

## Abstract

This work is a phytosociological approach to the montane rainforests of Peru with the aim of advancing on the diversity of plant communities, which we had already begun in previous research. From 364 phytosociological plots and 3389 species of the South American tropics, we have developed a cluster, using the Sørensen index, to know the similarities between the forests and their parallelism with bioclimatic conditions. After studying the existence of characteristic groups of the Peruvian forests, we have established different communities and phytosociological units for Peru. As a result, we have described seven associations, within three new alliances, which are gathered in the new order *Saurauio peruvianae-Condaminetalia corymbosae* of the new class *Morello pubescentis-Myrsinetea coriaceae*. In addition, two associations have been described within the class *Pruno rigidae-Oreopanacetea floribundae* (mesotropical laurel-like forests), and three for the class *Alnetea acuminatae* (alder forests and palm groves). The humid forests of Peru are closer to those of Ecuador and to those of the set formed by the three Colombian mountain ranges than to those of Bolivia and Argentina, due to the common flora these share with areas of Paraguay and even of the Parana River region.

## 1. Introduction

Tropical montane rainforests are located worldwide in areas where east–west winds (Trade Winds) blow against the mountains [1,2], establishing well-defined vegetation belts that depend on rainfall and humidity condensation at medium elevations.

In South America, winds from Amazonia push the evapotranspiration humidity to the Andean forests. The decrease in temperatures causes condensation of humidity, and consequently downpours, particularly between 1000 and 3500 m, producing forests of enormous diversity [3,4] between Venezuela and NW Argentina. They depend on rainfall oscillation of between 500 and 600 mm above 3500 m and more than 5000 mm at 600 m above sea level [5,6,7].

In the Peruvian Andes, some authors distinguish between mountain and brow forests [8,9,10,11]: the mountain extending between 1800 and 3400 m, where genera such as *Cecropia*, *Cinchona*, *Luehea*, and *Ochroma* are abundant, and are replaced by the mountain brow between 3400 and 3600 (3900) m above sea level, with *Ericaceae, Polylepis,* and *Weinmannia.* The most recent authors have established a more precise division [12,13,14]: mountain rainforests, between 600 and 650 and 1300–1400 m, where trees reach 35 m in height, palms abound but not *Mauritia*, and wild cocoa appears; cloud forests, between 1300 and 1400 m and 2500–2550 m, trees up to 20 m with tree ferns, where the most common trees belong to the genera *Brunellia, Ceroxylon*, *Hieronyma*, *Meliosma*, *Miconia*, *Myrcianthes*, *Saurauia*, and *Sapium*, and *Alnus acuminata* Kunth especially dominates along watercourses, and dwarf forests (mountain brow), between 2500 and 2550 and 3000–3800 m, up to 5–20 m of tree canopy, where *Axinaea*, *Cervantesia*, *Columellia*, *Drimys*, *Escallonia*, *Gaiadendron*, *Hedyosmum*, *Ilex*, *Miconia*, *Myrsine*, *Styrax*, *Symplocos*, and *Vallea* are the most common genera. Understories consist of scandent bamboos, mainly of the genus *Chusquea*, with vines of *Clematis*, *Mutisia*, *Jungia*, and *Passiflora*. The higher zones have forests of *Buddleja*, *Gynoxys*, and *Polylepis*. In general, mountain forests reach 4700 m in Bolivia, and 3800–4200 m from southern Peru to Venezuela. This division is very close to that of Malizia et al. [15] in Argentina, which distinguishes four vegetation belts: tropical premontane forest plots (<1500 m above sea level) associated with *Clarisia biflora* Ruiz & Pav., *Heliocarpus americanus* L., and *Solanum ochrophyllum* Van Heurck & Müll. Arg., while *Nectandra subbullata* Rohwer and *Piper obliquum* Ruiz & Pav. are related to tropical lower montane forest plots (i.e., 1500 to 2700 m above sea level). *Cornus peruviana* J.F.Macbr., *Hedyosmum scabrum* (Ruiz & Pav.) Solms, *Hesperomeles ferruginea* Lindl., *Licaria applanata* van der Werff, *Myrcianthes rhopaloides* (Kunth) McVaugh, *Myrsine dependens* (Ruiz & Pav.) Spreng., and *Podocarpus oleifolius* D.Don are associated with tropical upper forest plots (i.e., 2700 to 3500 m above sea level), while *Polylepis pauta* is grouped with plots above the upper forest line (>3500 m above sea level).

However, the montane rainforest also extends along the Pacific slope of the Andes between 2400 and 3800 m above sea level between Venezuela and northern Peru, with some relicts in the center, due to disturbances caused by the warm tropical current [16,17,18].

Among the 40,000 plant species recognized in the Andes, about 30,000 correspond to the mountain forests [19], and only about 5000 to the mountain forests of central Peru [20]. This enormous diversity is connected to the major uplift of the Central Andes during the Paleogene (65 to 34 Ma) and subsequent plate collisions that intensified mountain building in the Northern Andes (23 Ma) [21]. However, the Andes reached their modern elevation during the late–mid-Miocene (~12 Ma) and early Pliocene (~4.5 Ma) [22,23], which gave rise to the ecosystems we know today. This last period also saw the culmination of the formation of the Central American corridor [24], which led to the northward expansion of the Humboldt sea current, strengthening the aridity of western Peru and causing the disappearance of most of the settled forest masses. In addition, this phenomenon enabled the arrival of Laurasian taxa from families such as *Aquifoliaceae*, *Berberidaceae*, *Betulaceae*, *Cornaceae*, *Fagaceae*, and *Rosaceae*, which we find in the middle and upper levels of the Andes (bioclimatic meso-orotropical belts) linked to wind pollination on many occasions. Nevertheless, many of the families with tubuliform flowers which originated in Gondwana (i.e., *Acanthaceae*, *Apocynaceae*, *Bignoniaceae*, *Bromeliaceae*, *Ericaceae*, *Gesneriaceae*, *Musaceae*, *Palmae*) are more frequently found in the lower levels (thermo-infratropical belts), evidencing co-evolution with hummingbirds, nectar-feeding bats, and specialized birds. *Podocarpaceae* are relicts which originated in the South of the Andes, and which possibly arrived in the Pleistocene [25], forming meso-supratropical communities in cold and humid places, such as *Weinmannia*. The presence of Laurasian taxa such as *Alnus, Berberis, Cornus, Ilex, Quercus* (Colombia), and *Rubus* characterize the Neotropical montane forests [26], and separate them from those of Amazonia [11,12,27]. On the other hand, other authors include them as a biogeographical unit within Amazonia [5,28,29].

Although the phytosociological method of Braun-Blanquet [30] has been applied in numerous areas of the world (i.e., South Africa, Lesotho and Swaziland [31], American Arctic Zone [32], Western North America [33], Taiwan [34], Argentina [35], and the Caribbean region [36]), few phytosociological works on mountain rainforests in the extensive territory of South America have been published. Some examples are those of Venezuela [37,38,39], Colombia [40,41,42,43,44], Ecuador [45,46], Bolivia [47], and Argentina [48,49,50]. In Peru, only a few plots have been studied in some areas of the North [18]. Here, we present plots and plant communities of humid mountain forests throughout the country, comparing them to the adjacent territories, and carry out a syntaxonomical hierarchy assay, in addition to the published papers of the western arid desert [51,52,53] and the highlands [18,54].

## 2. Results

### 2.1. Relationships among South American Montane Rainforests

**Figure 1:**
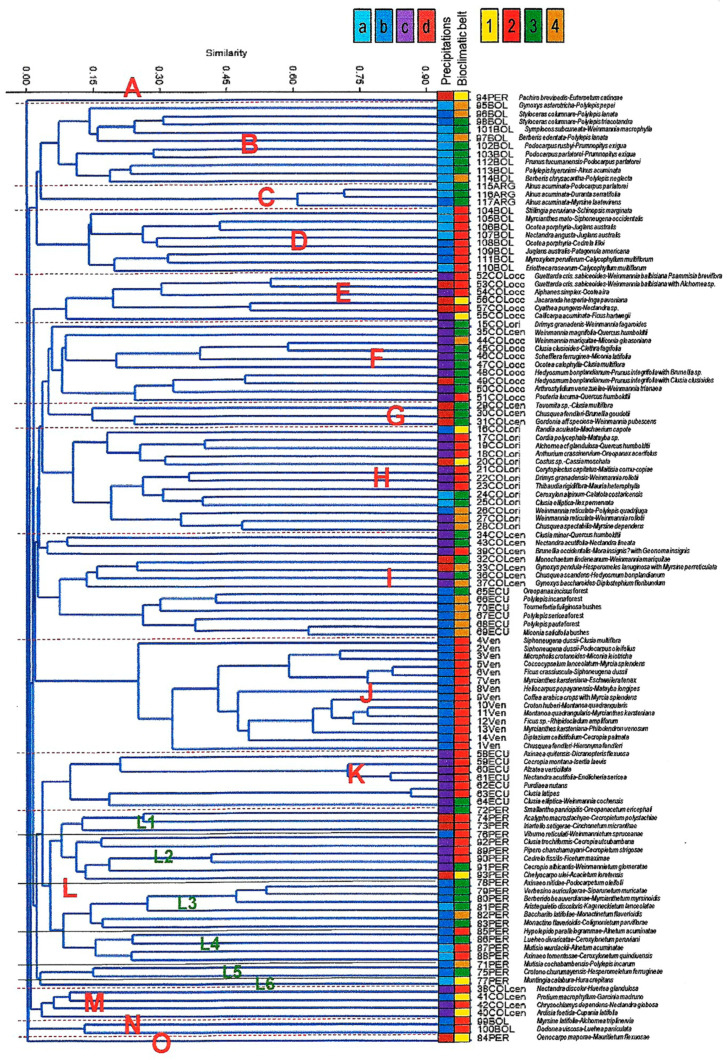
Cluster analysis with the unweighted pair-group average (UPGMA) applying the Sørensen index. Bioclimatic belts: 1: infratropical, 2: thermotropical, 3: mesotropical, 4: supratropical. Precipitation intervals: a: subhumid, b: humid, c: hyperhumid, d: ultra-hyperhumid. Capital letters are the groups detected in the dendrogram and explained in the text.

As a result of statistical analysis taking into consideration all the forests presented in the Supplementary Tables S1–S3, a dendrogram was generated (Figure 1). Here, we can distinguish 15 main groups: Groups A and O are greatly separated because they represent two very different forests from the Peruvian Amazonian lowlands: Group A containing *Euterpe catinga* Wallace (*Pachiro brevipedis-Euterpetum catingae* Galán de Mera 2001), and Group O containing *Mauritia flexuosa* L.f. (*Oenocarpo maporae-Mauritietum flexuosae* Galán de Mera 1996). Group B are the subhumid and humid meso-supratropical forests of Bolivia, and they are closely related to those of Argentina (C), because they contain common plants, such as *Alnus acuminata*, *Baccharis latifolia* (Ruiz & Pav.) Pers., *Dolichandra unguis-cati* (L.) L.G. Lohmann, *Duranta serratifolia* (Griseb.) Kuntze, *Podocarpus parlatorei* Pilg., *Rubus bogotensis* Kunth, and *Rubus boliviensis* Focke.

Group D are the thermotropical subhumid and humid forests of Bolivia, where we can also differentiate common taxa with that of Argentina such as *Juglans australis* Griseb., *Myrcianthes mato* (Griseb.) McVaugh, *Ocotea porphyria* (Griseb.) van der Werff, *Patagonula americana* L., *Schinopsis marginata* Engl., and *Siphoneugena occidentalis* D. Legrand. 

Between groups E to I, the montane rainforests of Colombia are found: Group E are the thermo- infratropical hyperhumid and ultra-hyperhumid forests of the western Andean mountain range with *Cyathea pungens* (Willd.) Domin, *Ficus hartwegii* Miq., *Jacaranda hesperia* Dugand, *Nectandra pichurim* (Kunth) Mez, *Ocotea ira* Mez & Pittier, and *Weinmannia balbisiana* Kunth. However, group F are especially mesotropical hyperhumid and ultra-hyperhumid forests in these western mountains where distinctive plants change to *Clethra fagifolia* Kunth, *Clusia clusioides* (Griseb.) D’Arcy, *Hedyosmum bonplandianum* Kunth, *Prunus integrifolia* (Sudw.) Sarg., *Quercus humboldtii* Bonpl., *Schefflera ferruginea* (Willd. ex Schult.) Harms, or *Weinmannia mariquitae* Szyszyl. The column 15COLori (*Drimys granadensis* L.f.-*Weinmannia fagaroides* Kunth from the Eastern Andean mountain range) is within group F because we find plant communities containing plants common with group F, such as *Dioscorea coriacea* Humb. & Bonpl. ex Willd., *Drimys granadensis*, *Myrsine dependens*, and *Sphyrospermum cordifolium* Benth. In the same way, 35COLcen (*Weinmannia magnifolia* Cuatrec.-*Quercus humboldtii* from the Central Andean mountain range) also contains plants common with the rest of the communities of group F, such as *Clusia multiflora* Kunth, *Escallonia myrtilloides* L.f., *Gaiadendron punctatum* (Ruiz & Pav.) G. Don, *Miconia latifolia* (D. Don) Naudin, *Palicourea angustifolia* Kunth, or *Uncinia hamata* (Sw.) Urb. Group G also includes three ultra-hyperhumid mesotropical plant communities from the Central Andean mountain range of Colombia, where there are species common with the occidental territories (*Alansmia lanigera* (Desv.) Moguel & M. Kessler, *Begonia umbellata* Kunth, *Clusia multiflora*, *Lophosoria quadripinnata* (J.F. Gmel.) C. Chr., *Miconia psychrophila* Naudin, and *Uncinia hamata*). Group H are mostly the branches belonging to the Eastern Andean mountain range of Colombia, where we can find some characteristic plants such as *Anthurium crassinervium* (Jacq.) Schott, *Cassia moschata* Kunth, *Cordia polycephala* (Lam.) I.M. Johnst., *Machaerium capote* Dugand, or *Thibaudia rigidiflora* A.C. Sm. for humid to ultra-hyperhumid infra- or thermo-tropical bioclimatic belts. In contrast, *Ceroxylon alpinum* Bonpl. ex DC.-*Calatola costaricensis* Standl. and *Clusia elliptica* Kunth-*Ilex pernervata* Cuatrec. are located in the mesotropical subhumid bioclimatic belt. 

Close to group H is group I, with columns from Central Colombia and Ecuador. The plant communities from Colombia are especially within hyperhumid and ultra-hyperhumid meso-supratropical bioclimatic belts. Here, we can again find species such as *Quercus humboldtii*, *Weinmannia mariquitae*, or *Hedyosmum humboldtianum*, but with characteristics such as *Chusquea spectabilis* L.G. Clark, *Clusia minor* L., *Polylepis quadrijuga* Bitter, or *Weinmannia reticulata* Ruiz & Pav. Humid supra-mesotropical branches of Ecuador reflect species common with Central Colombia such as *Drimys granadensis, Gaiadendron punctatum, Gaultheria myrsinoides* Kunth, *Miconia salicifolia* (Bonpl. ex Naudin) Naudin, *Oreopanax incisus* (Willd. ex Schult.) Decne. & Planch., *Vallea stipularis* L.f., or *Weinmannia pubescens* Kunth. 

Group J belongs to the Venezuelan columns with a humid thermotropical bioclimate where only widely distributed plants, such as *Anthurium nymphaeifolium* K. Koch & C.D. Bouché, *Blechnum occidentale* L., *Clusia multiflora*, *Myrcia splendens* (Sw.) DC., *Myrsine coriacea* (Sw.) R. Br. ex Roem. & Schult., or *Podocarpus oleifolius* are common with Colombia, Ecuador, and Peru.

Furthermore, group K are hyperhumid thermo- and mesotropical plant communities of Ecuador, where there are more plants common with Peru than in the case of group I. These include *Baccharis genistelloides* (Lam.) Pers., *Calceolaria calycina* Benth., *Cecropia angustifolia* Trécul, *Cyathea lechleri* Mett., *Gaultheria reticulata* Kunth, *Piper barbatum* Kunth, *Geonoma orbignyana* Mart., *Podocarpus oleifolius*, or *Rubus boliviensis*.

Group L represents the forests studied throughout Peru. Here, we can distinguish several subgroups: L1 are hyperhumid and ultra-hyperhumid thermo-mesotropical forests from Southern Peru, L2 are forests ranging from humid to hyperhumid and thermo- to mesotropical from Northern and Central Peru, L3 encompasses the subhumid to humid meso- and supratropical forests and shrub formations from Northern Peru, where *Oreopanax eriocephalus* Harms and *Baccharis latifolia* are respectively constant, subgroup L4 are the Andean alder forests that grow on hydromorphic soils with constant moisture, although they share a bioclimate that ranges from subhumid to humid and thermo- and mesotropical, L5 are high Andean hyperhumid meso- and supratropical forests from the South of Peru with *Polylepis incarum* (Bitter) M. Kessler & Schmidt-Leb. and *Hesperomeles ferruginea*, and finally, branch L6 is a unique plot containing *Muntingia calabura* L. and *Hura crepitans* L., a flooding forest within a subhumid infratropical bioclimate.

With group M, we return to the forests of the central Andean mountain range of Colombia, but here, there are the infra- and thermotropical humid and hyperhumid forests. These are located next to those of Peru due to common species such as *Disterigma acuminatum* (Kunth) Nied., *Dryopteris patula* (Sw.) Underw., *Miconia aggregata* Gleason, *Geonoma orbignyana*, *Hedyosmum racemosum* (Ruiz & Pav.) G. Don, *Piper arboreum* Aubl., *Serpocaulon fraxinifolium* (Jacq.) A.R. Sm., *Styloceras laurifolium* (Willd.) Kunth, and *Urera baccifera* (L.) Gaudich. ex Wedd. The same is applicable to the humid thermotropical plant communities *Myrsine latifolia* (Ruiz & Pav.) Spreng.-*Alchornea triplinervia* (Spreng.) Müll. Arg., and *Dodonaea viscosa* (L.) Jacq.-*Luehea paniculata* Mart. from Bolivia (group N), with species such as *Cedrela fissilis* Vell., *Morella pubescens* (Humb. & Bonpl. ex Willd.) Wilbur, *Myrsine coriacea*, *Myrsine latifolia*, and *Saurauia peruviana* Buscal. also present in Peru. Finally, group O is the *Oenocarpo maporae-Mauritietum flexuosae* association of palms from the ultra-hyperhumid infratropical Amazonian lowlands.

Using the Sørensen coefficient, a numerical similarity analysis of all forests for each country is presented in Table 1. Under each country name, the number of plots and their alpha diversity are presented.

Here, we can note that there is a shorter distance between the flora of the forests of Ecuador, Bolivia, and the western Colombian mountain range. However, the Venezuelan and Argentinean forests are at a greater distance, while the forests of Bolivia and Argentina are closer. In general, Peruvian forests are more similar to those of Ecuador and the three Colombian mountain ranges than Bolivian forests, because the latter have elements in common with Paraguay and the Parana region.

### 2.2. Phytosociological Study in Peru

Phytosociological results for the groups are shown in Appendix A (Table A1), which match those in the dendrogram (Figure 1).

#### 2.2.1. Describing the New Associations and Plant Communities

##### *Mutisia cochabambensis* Hieron.-*Polylepis incarum* community

Plot 214 (Ollachea, Puno, 3786 m above sea level, 13°46′13.04′′ S/70°30′13.92′′ W) (Supplementary Table S2). Diagnostic species: *Citharexylum dentatum* D. Don, *Elaphoglossum engelii* (H. Karst.) Christ, *Gaultheria vaccinioides* Griseb. ex Wedd., *Mutisia cochabambensis*, and *Polylepis incarum*.

Forest dominated by *Polylepis incarum*, humid supratropical, on rocky and stony areas, with a 40% slope and an average tree canopy of 10 m, spreading through the south of Peru.

##### *Smallantho parvicipitis-Oreopanacetum eriocephali* ass. nov.

Holotypus: Appendix A Table A2; Plot 215, Supplementary Table S2. Diagnostic species: *Begonia bracteosa* A. DC., *Canna iridiflora* Ruiz & Pav., *Croton regelianus* Müll. Arg., *Fuchsia boliviana* Carrière, *Melica scabra* Kunth, *Miconia aff. adinantha* Wurdack, *Pennisetum latifolium* Spreng., *Phenax angustifolius* (Kunth) Wedd., *Rubus boliviensis*, *Sambucus peruviana* Kunth, *Smallanthus parviceps* (S.F. Blake) H. Rob., and *Solanum aphyodendron* S. Knapp.

Hyperhumid mesotropical forests, with *Oreopanax eriocephalus*, installed on a steep slope (60%) and with a forest canopy of about 10 m, on deep sandy soils at the surface, susceptible to strong erosion. We have also studied this association in the San Gaban valley in southern Peru.

##### *Iriartello setigerae-Cinchonetum micranthae* ass. nov.

Holotypus: Appendix A Table A2; Plot 221, Supplementary Table S2. Diagnostic species: *Aphelandra aurantiaca* (Scheidw.) Lindl., *Cinchona micrantha* Ruiz & Pav., *Cyathea subincisa* (Kunze) Domin, *Dieffenbachia humilis* Poepp., *Heliconia hirsuta* L.f., *Iriartella setigera* (Mart.) H. Wendl., *Miconia lourteigiana* Wurdack, *Philodendron rudgeanum* Schott, *Ruagea insignis* (C. DC.) T.D. Penn., and *Smilax purhampuy* Ruiz.

Lower thermotropical ultra-hyperhumid forests very rich in Peruvian bark trees, with rainfall over 5000 mm. They are located in soft reliefs on clayey soils with a slope between 0% and 50%. The tree canopy is up to 30 m. They are spread throughout the South of Peru.

##### *Acalypho macrostachyae-Cecropietum polystachyae* ass. nov.

Holotypus: Appendix A Table A2; Plot 228, Supplementary Table S2. Diagnostic species: *Acalypha macrostachya* Jacq., *Cecropia polystachya* Trécul, and *Maranta arundinacea* L.

Upper thermotropical ultra-hyperhumid forests from Southern Peru, where rainfall reaches 3600 m. They are located in flat areas, but also on slopes of up to 70% on soils rich in clay, reaching a forest canopy of up to 30 m, where *Cecropia polystachya* becomes dominant.

##### *Crotono churumayensis-Hesperomeletum ferrugineae* ass. nov.

Holotypus: Appendix A Table A2; Plot 234, Supplementary Table S2. Diagnostic species: *Adiantum digitatum* Hook., *Baccharis genistelloides*, *Croton churumayensis* Croizat, *Cuphea cordata* Ruiz & Pav., *Gaultheria reticulata*, *Miconia alpina* Cogn., *Senna birostris* (Vogel) H.S. Irwin & Barneby, and *Tagetes elliptica* Sm.

Humid mesotropical forests with a tree canopy of about 8 m, from the south of Peru, that sit on ranker-type soils, stony on the surface, but with scant slope.

##### *Viburno reticulati-Weinmannietum spruceanae* ass. nov.

Holotypus: Appendix A Table A2; Plot 238, Supplementary Table S2. Diagnostic species: *Anthurium hamiltonii* Croat & Ligán, *Clusia crenata* Cuatrec., *Gleichenella pectinata* (Willd.) Ching, *Mandevilla fragrans* (Stadelm.) Woodson, *Pitcairnia paniculata* (Ruiz & Pav.) Ruiz & Pav., *Pouzolzia poeppigiana* (Wedd.) Killip, *Rubus urticifolius* Poir., *Viburnum reticulatum* (Ruiz & Pav. ex Oerst.) Killip, and *Weinmannia spruceana* Engl.

Humid thermotropical forests from Northern Peru (Cajamarca Region), on clayey soils of Tertiary volcanic origin and quaternary sediments with a slope up to 60%. The trees can reach a canopy of 20 m.

##### *Muntingia calabura*-*Hura crepitans* community

Plot 243 (Tamborapa, Cajamarca, 575 m above sea level, 5°25′44.42′′ S/78°57′39.38′′ W) (Supplementary Table S2). Diagnostic species: *Albizia multiflora* (Kunth) Barneby & J.W. Grimes, *Hura crepitans*, *Ichnanthus nemorosus* (Sw.) Döll, *Leucaena trichodes* (Jacq.) Benth., *Muntingia calabura*, *Paullinia alata* (Ruiz & Pav.) G. Don, *Piper nudilimbum* C. DC., *P. peltatum* L., and *Ruellia brevifolia* (Pohl) C. Ezcurra.

Forests overlooked by *Hura crepitans*, with a tree canopy of approximately 20 m, located on very clayey soils with periodic flooding under a sub-humid infratropical bioclimate, in areas near the Marañón River.

##### *Hypolepido parallelogrammae-Alnetum acuminatae* ass. nov.

Holotypus: Appendix A Table A2; Plot 278, Supplementary Table S2. Diagnostic species: *Acanthus ilicifolius* L., *Asplenium auriculatum* Sw., *Aulonemia longiaristata* L.G. Clark & Londoño, *Cyathea herzogii* Rosenst., *Cyathea lechleri*, *Geonoma orbignyana*, *Hedyosmum sprucei* Solms, *Hypolepis parallelogramma* (Kunze) C. Presl, *Hypolepis obtusata* (C. Presl) Kuhn, *Inga acuminata* Benth., *Miconia aff asperrima* Triana, *Miconia aggregata*, *Saccoloma inaequale* (Kunze) Mett., and *Serpocaulon fraxinifolium*.

Humid thermotropical alder forest, with tree ferns and a large number of shady ferns on black hydromorphic soils on gentle slopes (up to 40%). It forms a forest canopy of about 30 m in mountain areas south of the Huancabamba depression.

##### *Lueheo divaricatae-Ceroxylonetum peruviani* ass. nov.

Holotypus: Appendix A Table A2; Plot 284, Supplementary Table S2. Diagnostic species: *Austroeupatorium inulaefolium* (Kunth) R.M. King & H. Rob., *Ceroxylon peruvianum* Galeano, Sanín & K. Mejia, *Ladenbergia oblongifolia* (Humb. ex Mutis) L. Andersson, *Luehea divaricata* Mart., *Pueraria phaseoloides* (Roxb.) Benth., *Schistocarpha sinforosi* Cuatrec., *Styloceras laurifolium,* and *Viburnum incarum* Graebn.

Forest of the endemic palm *Ceroxylum peruvianum,* which is only distributed in the eastern Andes of the Amazonas department. It is a mesotropical humid forest that sits on hydromorphic soils near watercourses and slopes (20–40%) with permanent water. The tree canopy is about 20 m.

##### *Mutisio wurdackii-Alnetum acuminatae* ass. nov.

Holotypus: Appendix A Table A2; Plot 286, Supplementary Table S2. Diagnostic species: *Cortaderia jubata* (Lemoine ex Carrière) Stapf, *Mutisia wurdackii* Cabrera, and *Weinmannia costulata* Cuatrec.

Alder forest in northern Peru, found in permanent water courses forming a gallery of trees that reach 15 m canopy, under a subhumid thermotropical bioclimate.

##### *Axinaeo tomentosae-Ceroxylonetum quindiuensis* ass. nov.

Holotypus: Appendix A Table A2; Plot 289, Supplementary Table S2. Diagnostic species: *Axinaea tomentosa* Cogn., *Cedrela odorata* L., *Ceroxylon quindiuense* (H. Karst) H. Wendl., *Citronella incarum* (J.F. MacBr.) R.A. Howard, *Cornus peruviana*, *Cronquistianthus chachapoyensis* R.M. King & H. Rob., *Erythrina aff edulis* Micheli, *Hedyosmum lechleri* Solms, *Vismia pozuzoensis* Engl., *Solanum barbulatum* Zahlbr., and *Viburnum pichinchense* Benth.

Palm grove dominated by *Ceroxylon quindiuense,* whose canopy is up to 30 m. It sits on peaty soil under a subhumid thermotropical bioclimate with a flat or slightly concave relief. It is an endemic association of the Eastern Andes in the departments of Amazonas and Cajamarca.

##### *Piperi chanchamayani-Cecropietum strigosae* ass. nov.

Holotypus: Appendix A Table A2; Plot 293, Supplementary Table S2. Diagnostic species: *Adiantum raddianum* C. Presl, *Ageratina tambillensis* (Hieron.) R.M. King & H. Rob., *Anthurium grande* W. Bull, *Canna paniculata* Ruiz & Pav., *Cavendishia punctata* (Ruiz & Pav. Ex J. St. Hil.) Sleumer, *Cecropia angustifolia, Cecropia strigosa* Trécul, *Centropogon altus* E. Wimm., *Chromolaena leptocephala* (DC.) R.M. King & H. Rob., *Coriaria myrtifolia* L., *Meriania tomentosa* (Cogn.) Wurdack, *Miconia sanguinea* (D. Don) Triana, *Pilea pavonii* Wedd., *Psammisia coarctata* (Ruiz & Pav.) A.C. Sm., *Solanum asperolanatum* Ruiz & Pav., *Solanum mite* Ruiz & Pav., and *Thelypteris euchlora* (Sodiro) C.F. Reed.

Hyperhumid upper thermotropical forest with *Cecropia* from central Peru (Chanchamayo area) developing on clay-rich soils, with slopes of up to 40% and a tree canopy of up to 25 m.

##### *Cedrelo fissilis-Ficetum maximae* ass. nov.

Holotypus: Appendix A Table A2; Plot 298, Supplementary Table S2. Diagnostic species: *Alsophila mostellaria* Lehnert, *Begonia parviflora* Poepp. & Endl., *Campyloneurum ophiocaulon* (Klotzsch) Fée, *Centropogon granulosus* C. Presl, *Columnea inaequilatera* Poepp. & Endl., *Croton lechleri* Müll. Arg., *Ficus maxima* Mill., *Heliconia subulata* Ruiz & Pav., *Inga adenophylla* Pittier, *Lastreopsis effusa* (Sw.) Tindale, *Nephrolepis pectinata* (Willd.) Schott, *Ocotea obovata* (Ruiz & Pav.) Mez, *Pteris altissima* Poir., *Selaginella trisulcata* Aspl., *Serjania communis* Cambess., *Serpocaulon loriceum* (L.) A.R. Sm., *Prestoea carderi* (W. Bull) Hook. f., *Thelypteris jamesonii* (Hook.) R.M. Tryon, *Thelypteris salzmannii* (Fée) C.V. Morton, *Vernonanthura patens* (Kunth) H. Rob., *Urera baccifera,* and *Urera verrucosa* (Liebm.) V.W. Steinm.

Hyperhumid thermotropical forest of central Peru periodically flooded with clayey soils with certain hydromorphism. It is located on gentle slopes (0–40%). *Ficus* and *Cedrela* are prominent in the canopy, reaching about 20 m.

##### *Cecropio albicantis-Weinmannietum glomeratae* ass. nov.

Holotypus: Appendix A Table A2; Plot 300, Supplementary Table S2. Diagnostic species: *Ageratina rhytidodes* (B.L. Rob.) R.M. King & H. Rob., *Bomarea pseudopurpurea* Hofreiter & E. Rodr., *Cavendishia nobilis* Lindl., *Cecropia albicans* Trécul, *Centropogon hirtus* (G. Don) C. Presl, *Centropogon reflexus* C. Presl, *Dioscorea mitoensis* R. Knuth, *Disterigma acuminatum, Elleanthus aurantiacus* (Lindl.) Rchb. f., *Miconia galactantha* Naudin, *Piper augustum* Rudge, *Sticherus revolutus* (Kunth) Ching, *Thelypteris pachyrhachis* (Kunze ex Mett.) Ching, *Tibouchina lepidota* (Bonpl.) Baill., *Tibouchina saxosa* Gleason, *Vaccinium crenatum* (G. Don) Sleumer, *Weinmannia auriculata* D. Don, *Weinmannia latifolia* C. Presl, and *Weinmannia microphylla* Kunth.

Hyperhumid mesotropical forest of central Peru rich in *Weinmannia* species, with a forest canopy of 10–15 m, located on very clayey soils, reddened by iron oxides, with a relief of steep slopes (40–60%).

##### *Clusia trochiformis-Cecropia utcubambana* Community

Plot 305 (Below Carpish, Huánuco, 1762 m above sea level, 9°28′02.02′′ S/76°00′02.93′′ W) (Supplementary Table S2). Diagnostic species: *Barnadesia corymbosa* (Ruiz & Pav.) D. Don, *Canna bangii* Kraenzl., *Dryopteris patula, Fuchsia macrophylla* I.M. Johnst., *Margaritopsis boliviana* (Standl.) C.M. Taylor, *Palicourea angustifolia, Piper semicordulatum* Trel., *Schefflera pentandra* (Pav.) Harms, and *Serpocaulon adnatum* (Kunze ex Klotzsch) A.R. Sm. 

Hyperhumid lower thermotropical forest dominated by *Cecropia utcubambana* that reaches a height of about 20 m. We have only been able to carry out one plot on brown clay soils with a steep slope (50%).

##### *Chelyocarpo ulei-Acacietum loretensis* ass. nov.

Holotypus: Appendix A Table A2; Plot 306, Supplementary Table S2. Diagnostic species: *Acacia loretensis* J.F. Macbr., *Acalypha stricta* Poepp., *Banara guianensis* Aubl., *Bauhinia tarapotensis* Benth., *Boehmeria pavonii* Wedd, *Chelyocarpus ulei* Dammer, *Clitoria pozuzoensis* J.F. Macbr., *Corytoplectus speciosus* (Poepp.) Wiehler, *Heliconia rostrata* Ruiz & Pav., *Miconia amplexicaulis* Naudin, *Paullinia serjaniifolia* Triana & Planch., *Perebea angustifolia* (Poepp. & Endl.) C.C. Berg, *Piper arboreum*, *Polybotrya caudata* Kunze, *Selaginella haematodes* (Kunze) Spring, *Sloanea ptariana* Steyerm., *Tectaria antioquoiana* (Baker) C. Chr., and *Thelypteris patens* (Sw.) Small.

Ultra-hyperhumid infratropical Amazon forests, from the lowest parts, with a hilly relief of the Peruvian Andes (800 m above sea level). They are settled on very yellowish silty soils, with slopes of up to 50%, and a tree canopy of about 30 m. The presence of the palm *Chelyocarpus ulei* indicates the Amazonian character of this forest, as does *Heliconia rostrata* and wild forms of *Theobroma cacao* L.

#### 2.2.2. Describing New Alliances, Orders and Classes

With this work, we have been able to extend the forest communities with Andean alders. In the north of Peru, it is differentiated with the *Cyatheo herzogii-Alnion acuminatae* all. nov., the montane thermotropical forests on clayey soils with hydromorphism–holotypus alliance: *Hypolepido parallelogrammae-Alnetum acuminatae* ass. nov.; diagnostic species: The same as in the association. On the other hand, the other associations (*Lueheo divaricatae-Ceroxylonetum peruviani* ass. nov., *Mutisio wurdackii-Alnetum acuminatae* ass. nov., and *Axinaeo tomentosae-Ceroxylonetum quindiuensis* ass. nov.), which are riverside and peatland forests from Northern Peru, are brought together in the *Escallonio pendulae-Alnion acuminatae* all. nov.–holotypus alliance: *Axinaeo tomentosae-Ceroxylonetum quindiuensis* ass. nov.; diagnostic species: *Bia alienata* Didr., *Escallonia pendula* (Ruiz & Pav.) Pers., *Ladenbergia oblongifolia, Pentacalia reflexa* (Kunth) Cuatrec., *Thelypteris pennata* (Poir.) C.V. Morton, and *Styloceras laurifolium.* All these alder forest alliances belong to the order *Alnetalia acuminatae* ord. nov. and the class *Alnetea acuminatae* Galán de Mera 2005 (Appendix A, Table A1, blue square).

The ultra-hyperhumid montane forests of southern Peru are joined together in the *Serpocaulo dasypleuronis-Alchorneion latifoliae* all. nov. (Appendix A, Table A1, orange square)–holotypus alliance: *Iriartello setigerae-Cinchonetum micranthae* ass. nov.; diagnostic species: *Alchornea latifolia* Sw., *Cyathea delgadii* Sternb., *Graffenrieda cucullata* (Triana) L.O. Williams, *Lasiacis ligulata* Hitchc. & Chase, *Olyra latifolia* L., *Psychotria poeppigiana* Müll. Arg., *Serpocaulon dasypleuron* (Kunze) A.R. Sm., and *Trema micrantha* (L.) Blume, while those in the north are included in the *Pteridi creticae-Cyatheion caracasanae* all. nov. (Appendix A, Table A1, pink square)–holotypus alliance: *Viburno reticulati-Weinmannietum spruceanae* ass. nov.; diagnostic species: *Begonia peruviana* A. DC., *Cecropia utcubambana* Cuatrec., *Cyrtocymura scorpioides* (Lam.) H. Rob., *Fuchsia mathewsii* J.F. Macbr., *Miconia adinantha, Pennisetum peruvianum* Trin., *Piper lineatum* Ruiz & Pav., *Pteris cretica* L., and *Seemannia sylvatica* (Kunth) Baill. The forests of central Peru belong to the *Sanchezio oblongae-Hedyosmion racemosi* all. nov. (Appendix A, Table A1, green square)–holotypus alliance: *Cecropio albicantis-Weinmannietum glomeratae* ass. nov.; diagnostic species: *Anthurium breviscapum* Kunth, *Baccharis decussata subsp. jelskii* (Hieron.) Joch. Müll., *Blepharodon salicinus* Decne., *Cedrela fissilis, Clusia trochiformis* Vesqne, *Dennstaedtia auriculata* H. Navarrete & B. Øllg., *Diplazium tungurahuae* (Sodiro) C. Chr., *Dryopteris wallichiana* (Spreng.) Hyl., *Fuchsia ovalis* Ruiz & Pav., *Hedyosmum racemosum, Geonoma stricta* (Poit.) Kunth, *Gurania lobata* (L.) Pruski, *Iresine diffusa* Humb. & Bonpl. ex Willd., *Liabum nudicaule* H. Rob., *Miconia affinis* DC., *Miconia cyanocarpa* Naudin, *Monnina marginata* C. Presl, *Monstera obliqua* Miq., *Niphidium albopunctatissimum* Lellinger, *Oreopanax polycephalus* Harms, *Palicourea guianensis* Aubl., *Passiflora rubra* L., *Pilea haenkei* Killip, *Piper chanchamayanum* Trel., *Piper malifolium* Trel., *Pteris podophylla* Sw., *Pteris quadriaurita* Retz., *Sanchezia oblonga* Ruiz & Pav., *Weinmannia glomerata* C. Presl, and *Zeugites americanus* Willd.

The Peruvian alliances can be gathered in the new order *Saurauio peruvianae-Condaminetalia corymbosae* ord. nov.–holotypus order: *Sanchezio oblongae-Hedyosmion racemosi* all. nov.; diagnostic species: *Ageratina sternbergiana* (DC.) R.M. King & H. Rob., *Alchornea glandulosa* Poepp., *Baccharis inamoena* Gardner, *Begonia cyathophora* Poepp. & Endl., *Blechnum cordatum* (Desv.) Hieron., *Blechnum occidentale, Cavendishia bracteata* (Ruiz & Pav. ex J. St. Hil) Hoerold, *Cinchona pubescens* Vahl, *Condaminea corymbosa* (Ruiz & Pav.) DC., *Conostegia inusitata* Wurdack, *Ctenitis sloanei* (Poepp. ex Spreng.) C.V. Morton, *Cyathea caracasana* (Klotzsch) Domin, *Dioscorea altissima* Lam., *Disterigma alaternoides* (Kunth) Nied., *Heliocarpus americanus, Morella pubescens, Munnozia hastifolia* (Poepp.) H. Rob. & Brettell, *Myriocarpa stipitata* Benth., *Myrsine coriacea, Nephrolepis cordifolia* (L.) C. Presl, *Niphidium crassifolium* (L.) Lellinger, *Ochroma pyramidale* (Cav. ex Lam.) Urb., *Palicourea amethystina* (Ruiz & Pav.) DC., *Polystichum montevidense* (Spreng.) Rosenst., *Pteridium arachnoideum* (Kaulf.) Maxon, *Saurauia peruviana, Schefflera acuminata* (Pav.) Harms, *Serpocaulon caceresii* (Sodiro) A.R. Sm., *Siparuna aspera* (Ruiz & Pav.) A. DC., *Smilax domingensis* Willd., *Tradescantia cymbispatha* C.B. Clarke, and *Vismia tomentosa* Ruiz & Pav., and in the new phytosociological class *Morello pubescentis-Myrsinetea coriaceae* cl. nov., whose diagnostic species are those of the order. Both order and class may exceed the territory of Peru, judging by the species common with Venezuela, Colombia, Ecuador, Bolivia, and Argentina.

## 3. Discussion

### 3.1. Relationships among South American Montane Rainforests

According to Table 1, the mountain forests of Peru bear a greater resemblance from a floristic point of view to those of Ecuador and to the sum of the entire Colombian Andes than to those of Bolivia. However, as shown in the dendrogram in Figure 1, the forests of Peru form a well-defined unit, probably because they are confined between the Huancabamba depression in the north and the Abancay deflection in the south [55]. The depression of Huancabamba separates the north from the center of the Andes, while in the deflection of Abancay, the eastern cordillera begins a granitic arch from the basin of the Urubamba River towards the South [56], with the characteristic forests of the alliance *Serpocaulo dasypleuronis*-*Alchorneion latifoliae* all. nov. However, many characteristic species of the new class *Morello pubescentis-Myrsinetea coriaceae* cl. nov. are also found in Ecuador and Colombia (Appendix A, Table A1), so this class could go beyond the Peruvian Andes. On the other hand, we do not know of a phytosociological class from the humid–ultrahyperhumid Andes of Ecuador [46,57]. In the Eastern Cordillera of Colombia, the class *Palicoureo leuconerae-Cybianthetea iteoides* Rangel, Cleef & Arellano 2008 has been described, but without following the precepts of the Code [44]. In a previous paper [18], we had established the class Nectandro laurel-Licarietea cannellae Izco 2013 [57] in Peru, and although some of its characteristics, such as *Licaria canella* (Meisn.) Kosterm., *Isertia laevis* (Triana) Boom, or *Guzmania killipiana* L.B. Sm., exist in Peru, its presence is not evident in our plots.

The forests of Bolivia are different from those of Peru, due to the elements common with the forests to the south of the Amazonian basin, in Brazil, Paraguay, and Argentina, such as *Inga saltensis* Burkart, *Juglans australis*, *Myrcianthes mato*, *Ocotea porphyria*, *Podocarpus parlatorei*, and *Schinopsis brasiliensis* Engl. [47], and the forests of Argentina, even those of the Parana basin, such as *Allophylus edulis* (A. St.-Hil., A. Juss. & Cambess.) Radlk., *Baccharis coridifolia* DC., *Blepharocalyx salicifolius* (Kunth) O. Berg, *Myrsine laetevirens* (Mez) Arechav., or *Sebastiania commersoniana* (Baill.) L.B. Sm. & Downs [35,50].

### 3.2. Phytosociological Units Previously Described

According to Appendix A, Table A1, the rainforest with *Polylepis—Mutisia cochabambensis-Polylepis incarum* community—of Southern Peru belong to the *Polylepidion incano*-*besseri* Navarro in Navarro & Maldonado 2002 alliance, the Andean *Polylepidetalia racemosae* Galán de Mera & Cáceres in Galán de Mera, Rosa & Cáceres 2002 order, and *Polylepidetea tarapacano-besseri* Rivas-Martínez & Navarro in Navarro & Maldonado 2002 class [58,59]. In Ecuador, we also find *Polylepis* forests that belong to the same class [45], but the phytosociological units described with *Polylepis incana* Kunth, *Polylepis pauta* Hieron., and *Polylepis sericea* Wedd., do not follow the Code, although they clearly constitute different associations with the Peruvian ones. The eastern associations in Bolivia also constitute different communities from those of Peru, as they are dominated by *Polylepis besseri* Hieron. [60], absent from Peru, and *Polylepis tomentella* Wedd., which forms forests both in Bolivia [61] and in eastern areas of Peru [62], where other associations could be described.

Classes *Pruno rigidae-Oreopanacetea floribundi* Galán de Mera 2005 (Appendix A, Table A1, red square) and *Clematido peruvianae-Baccharitetea latifoliae* Galán de Mera, Sánchez Vega, Montoya, Linares, Campos & Vicente 2015 (Appendix A, Table A1, grey square) are well represented in at least subhumid areas of the Peruvian Andes. *Pruno-Oreopanacetea* includes the new associations *Smallantho parvicipitis-Oreopanacetum eriocephali* ass. nov. and *Crotono churumayensis-Hesperomeletum ferrugineae* ass. nov., and the previously described *Axinaeo nitidae-Podocarpetum oleifolii* Galán de Mera, Sánchez Vega, Montoya, Linares, Campos & Vicente 2015—north western Peruvian humid-hyperhumid mesotropical laurel-like forest, *Verbesino auriculigerae-Siparunetum muricatae* Galán de Mera, Sánchez Vega, Montoya, Linares, Campos & Vicente 2015—humid-hyperhumid mesotropical laurel-like forest of anthropic origin by disturbance of the previous one, *Berberido beauverdianae-Myrcianthetum myrsinoidis* Galán de Mera, Sánchez Vega, Montoya, Linares, Campos & Vicente 2015—north western Peruvian subhumid-humid mesotropical forest: they are found at higher altitudes and lower humidity, in contact with *Podocarpus oleifolius* forests, and *Aristeguietio discoloris-Kageneckietum lanceolatae* Galán de Mera, Sánchez Vega, Montoya, Linares, Campos & Vicente 2015—dry-subhumid upper mesotropical forests of the western Andes of Northern Peru. Although *Smallantho parvicipitis-Oreopanacetum eriocephali* ass. nov. and *Crotono churumayensis-Hesperomeletum ferrugineae* ass. nov. are eastern associations, we consider them part of the *Monnino pilosae-Myrcianthion myrsinoidis* Galán de Mera, Sánchez Vega, Montoya, Linares, Campos & Vicente 2015 alliance and the *Cestro auriculati-Prunetalia rigidae* Galán de Mera & Rosa in Galán de Mera, Rosa & Cáceres 2002 order, despite being less rich in characteristics [18], since both *Monnina pilosa* Kunth and *Myrcianthes myrsinoides* (Kunth) Grifo are widely distributed on the eastern Andean slopes.

The *Clematido peruvianae-Baccharitetea latifoliae* class includes shrub communities (Appendix A, Table A1, grey square), which constitute the succession of the *Pruno rigidae-Oreopanacetea floribundi* class in wet areas. Here, we have studied the previously described associations *Baccharito latifoliae-Monactinetum flaverioidis* Galán de Mera, Sánchez Vega, Montoya, Linares, Campos & Vicente 2015 and *Monactino flaverioidis-Colignonietum parviflorae* Galán de Mera, Sánchez Vega, Montoya, Linares, Campos & Vicente 2015. Both are from subhumid–humid areas, but the first is a supratropical association while the second is mesotropical, and they are part of the alliance *Otholobio munyensis-Rubion robusti* Galán de Mera, Sánchez Vega, Montoya, Linares, Campos & Vicente 2015 [18], which replaces *Mutisio acuminatae-Ophryosporion peruvianae* Galán de Mera & Cáceres in Galán de Mera, Rosa & Cáceres 2002 in northern Peru [58], and *Saturejion bolivianae* Seibert & Menhofer 1991 in eastern Bolivia [63]. All these alliances are included in the order *Mutisio acuminatae-Baccharitetalia latifoliae* Galán de Mera & Cáceres in Galán de Mera, Rosa & Cáceres 2002.

In our study, there are three Amazonian associations (Appendix A, Table A1, columns 14, 23, and 24), the new association *Chelyocarpo ulei-Acacietum loretensis* and the previously described *Oenocarpo maporae-Mauritietum flexuosae* Galán de Mera 1996 [64] and *Pachiro brevipedis-Euterpetum catingae* Galán de Mera 2001 [65]. We have included the sub-Andean *Chelyocarpo ulei-Acacietum loretensis* within *Morello pubescentis-Myrsinetea coriaceae* cl. nov., although it has certain similarities with the communities of the Colombian sub-Andean class *Smilaco floribundae-Ingetea edulis* Rangel, Cleef & Salamanca in Rangel, Cleef, Salamanca & Ariza 2005 [43], especially due to the presence of *Smilax domingensis* Willd. and *Inga edulis* Mart. However, species such as *Cnemidaria quitensis* (Domin) R.M. Tryon, *Otoba lehmannii* (A.C. Sm.) A.H. Gentry, *Ossaea bracteata* Triana, *Schefflera bejucosa* Cuatrec., or *Wettinia radiata* (O.F. Cook & Doyle) R. Bernal were not found in the Peruvian forests. The palm groves *Oenocarpo-Mauritietum* and *Pachiro-Euterpetum* are edapho-hygrophilous associations of the ultra-hyperhumid infratropical belt of the lower Amazonia. Their floristic composition is very different with respect to the class *Morello pubescentis-Myrsinetea coriaceae*, since the palms *Oenocarpus bataua* Mart., *Oenocarpus mapora* H. Karst., *Mauritia flexuosa*, and *Euterpe catinga* become dominant, as well as in other forests of the Colombian Amazonia [66], and in the Orinoco plains of Venezuela [67] and Colombia [68]; therefore, they surely constitute a particular class.

### 3.3. Syntaxonomical Checklist for the Montane Rainforests of Peru

This scheme was ordered according to the plant communities and groups of Appendix A, Table A1.

*Polylepidetea tarapacano-besseri* Rivas-Martínez & Navarro in Navarro & Maldonado 2002

*Polylepidetalia racemosae* Galán de Mera & Cáceres in Galán de Mera, Rosa & Cáceres 2002

*Polylepidion incano-besseri* Navarro in Navarro & Maldonado 2002

*Mutisia cochabambensis-Polylepis incarum* community

*Pruno rigidae-Oreopanacetea florifundae* Galán de Mera 2005

*Cestro auriculati-Prunetalia rigidae* Galán de Mera & Rosa in Galán de Mera, Rosa & Cáceres 2002

*Monnino pilosae-Myrcianthion myrsinioidis* Galán de Mera, Sánchez Vega, Montoya, Linares, Campos & Vicente 2015

*Axinaeo nitidae-Podocarpetum oleifolii* Galán de Mera, Sánchez Vega, Montoya, Linares, Campos & Vicente 2015

*Verbesino auriculigerae-Siparunetum muricatae* Galán de Mera, Sánchez Vega, Montoya, Linares, Campos & Vicente 2015

*Berberido beauverdianae-Myrcianthetum myrsinoidis* Galán de Mera, Sánchez Vega, Montoya, Linares, Campos & Vicente 2015

*Aristeguietio discoloris-Kageneckietum lanceolatae* Galán de Mera, Sánchez Vega, Montoya, Linares, Campos & Vicente 2015

*Smallantho parvicipitis-Oreopanacetum eriocephali* ass. nov.

*Crotono churumayensis-Hesperomeletum ferrugineae* ass. nov.

*Clematido peruvianae-Baccharitetea latifoliae* Galán de Mera, Sánchez Vega, Montoya, Linares, Campos & Vicente 2015

*Mutisio acuminatae-Baccharitetalia latifoliae* Galán de Mera & Cáceres in Galán de Mera, Rosa & Cáceres 2002

*Otholobio munyensis-Rubion robusti* Galán de Mera, Sánchez Vega, Montoya, Linares, Campos & Vicente 2015

*Baccharito latifoliae-Monactinetum flaverioidis* Galán de Mera, Sánchez Vega, Montoya, Linares, Campos & Vicente 2015

*Monactino flaverioidis-Colignonietum parviflorae* Galán de Mera, Sánchez Vega, Montoya, Linares, Campos & Vicente 2015

*Alnetea acuminatae* Galán de Mera 2005

*Alnetalia acuminatae* Galán de Mera & Rosa in Galán de Mera, Rosa & Cáceres 2002

*Cyatheo herzogii-Alnion acuminatae* all. nov.

*Hypolepido parallelogrammae-Alnetum acuminatae* ass. nov.

*Escallonio pendulae-Alnion acuminatae* all. nov.

*Lueheo divaricatae-Ceroxylonetum peruviani* ass. nov.

*Mutisio wurdackii-Alnetum acuminatae* ass. nov.

*Axinaeo tomentosae-Ceroxylonetum quindiuensis* ass. nov.

*Morello pubescentis-Myrsinetea coriaceae* cl. nov.

*Saurauio peruvianae-Condaminetalia corymbosae* ord. nov.

*Serpocaulo dasypleuronis-Alchorneion latifoliae* all. nov.

*Iriartello setigerae-Cinchonetum micranthae* ass. nov.

*Acalypho macrostachyae-Cecropietum polystachyae* ass. nov.

*Pteridi creticae-Cyatheion caracasanae* all. nov.

*Viburno reticulati-Weinmannietum spruceanae* ass. nov.

*Muntingia calabura-Hura crepitans* community

*Sanchezio oblongae-Hedyosmion racemose* all. nov.

*Piperi chanchamayani-Cecropietum strigosae* ass. nov.

*Cedrelo fissilis-Ficetum maximae* ass. nov.

*Cecropio albicantis-Weinmannietum glomeratae* ass. nov.

*Clusia trochiformis-Cecropia utcubambana* community

*Chelyocarpo ulei-Acacietum loretensis* ass. nov.

Insertae sedis

*Oenocarpo maporae-Mauritietum flexuosae* Galán de Mera 1996

*Pachiro brevipedis-Euterpetum catingae* Galán de Mera 2001

## 4. Materials and Methods

### 4.1. Study Area

**Figure 2:**
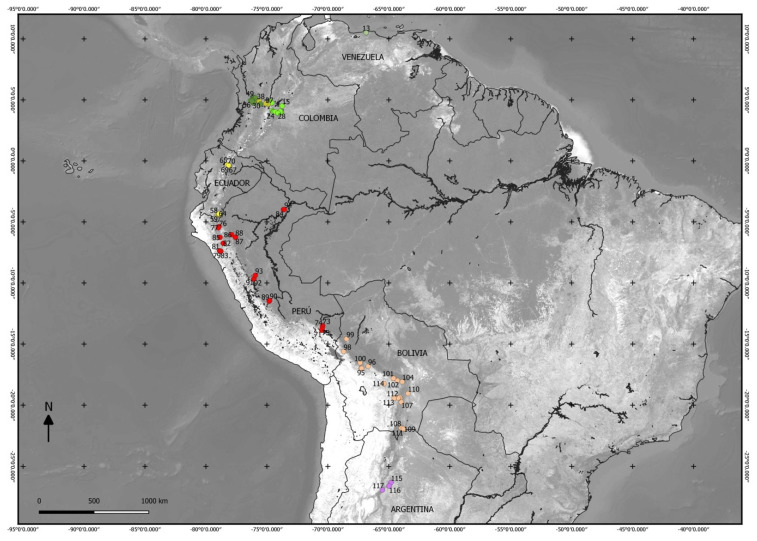
Map of tropical South America with the areas studied in Peru and surrounding countries. Numbers of dots correspond to those of Supplementary Tables S1 and S3. Map generated by Quantum Geographic Information System (QGIS) 3.0.1 software [69].

The mountain rainforests of the western and eastern slopes of the Peruvian Andes were studied (4°29′34.1′′ S to 14°37′52.68′′ S): on the north, those located in the vicinity of the Huancabamba depression (5°48′0 S), and on the south, those located in the surroundings of the Abancay deflection (13°16′37.22′′ S) [55], which are joined to those of central Peru (Figure 2).

The forests were studied at altitudes between 575 and 3786 m above sea level, with some of them located in lowland Amazonian forest at an elevation between 120 and 150 m. In general, they are situated in the eastern Andean mountain range, on Paleozoic granite and metamorphic rocks, although Jurassic limestone outcrops are frequent. However, in the north, Tertiary volcanism abounds in the western territories, as well as Quaternary sediments and marine volcanic sedimentary facies in the area of the Marañón River [70].

From a biogeographical point of view, Peruvian montane rainforests belong to Yungenian Province (Tropical Subandean Region, Neotropical-Austroamerican Kingdom) with a tropical pluvial bioclimate [27]. To examine altitudinal relationships between climate and forest plant communities, the bioclimatic system of Rivas-Martínez [71] was applied, using data for at least 30 years [72], from several meteorological stations as near as possible to the studied forests using the location coordinates of the forests and the stations found on Google Earth Pro© (Supplementary Table S1). We used this method because it precisely reflects the correspondence between bioclimatic belts and vegetation associations.

This bioclimatic model is based on thresholds of the Thermicity index (It) versus the intervals of annual precipitations (P in mm) (we did not use the humidity index (Io) because results do not reflect a vegetation type on some occasions) coincident with altitudinal and latitudinal areas of flora and vegetation, called bioclimatic belts. The Thermicity index is based on mean annual temperature (T in °C), and average maximum (M) and minimum (m) temperatures of the coldest month (It = (T + M + m) 10). Bioclimatic belts coincide with natural plant associations, and for Peru, six bioclimatic belts were defined and mapped [73]: Infratropical (It > 690), thermotropical (It 490 to 690), mesotropical (It 320 to 490), supratropical (It 160 to 320), orotropical (It 50 to 160), and cryorotropical (It < 50). P (in mm) intervals are the following: Ultra-hyperarid (P < 5), hyperarid (5 to 30), arid (31 to 100), semiarid (101 to 300), dry (301 to 500), subhumid (501 to 900), humid (901 to 1500), hyperhumid (1501 to 2500), and ultra-hyperhumid (>2500).

### 4.2. Plots and Flora of Peru

As it is very difficult to calculate the minimum phytosociological area in a mountain rainforest to carry out vegetation plots, we have established plots of 100 m^2^, as proposed by Dengler [74], up to 0.1 ha, as in Gentry’s methods, according to the forest complexity from a subhumid area to an ultra-hyperhumid area. As Gentry pointed out, the 0.1 ha transect method is ideally suited to collect data from multiple sites, in order to generate comparative data on the taxonomic composition in rainforests [75].

To identify the flora of the plots, we especially used some specific systematic works [76,77,78,79,80,81,82,83,84,85], floristic catalogues [86,87,88], and the herbaria COL (National University of Colombia), CPUN (National University of Cajamarca), CUZ (National University of San Antonio Abad of Cuzco), F (Field Museum of Natural History), MO (Missouri Botanical Garden), NY (The New York Botanical Garden), P (Natural History Museum of Paris), US (Smithsonian Institution), and USM (Natural History Museum of the Mayor University of San Marcos) (acronyms according to Thiers [89]).

Plant names were updated using the database The Plant List [90].

### 4.3. Relationships among South American Rainforests

Taking a total of 364 plots and 3389 species, we built a matrix (Supplementary Table S2), in which 106 plots were carried out by the authors in Peru, and 258 came from the bibliography: 14 from Venezuela [38], 96 from Colombia [41,42,43,44], 103 from Ecuador [45,46], 24 from Bolivia [47], and 21 from Argentina [48] (Figure 2). To obtain a synthetic matrix (Supplementary Table S3) grouping species, we performed a cluster analysis with the unweighted pair-group average (UPGMA) using the Sørensen index [91] in order to observe the similarity between the columns and their linkage with precipitation intervals and bioclimatic belts. To find a numerical similarity between the mountain rainforest flora, we synthesized all plots into a single column per country with JUICE software [92], and then computed the Sørensen index. UPGMA and Sørensen index were performed using PAST 4.03 [93] software.

### 4.4. Vegetation Classification

We used the phytosociology method of Braun-Blanquet [30] for vegetation classification, the aim of which is to define vegetation units by grouping plots with similar species compositions together and arrange these units into a hierarchical system for comparing the qualitative and quantitative floristic compositions of different geographic spaces. 

To group characteristic species into phytosociological units, and to identify fidelity among plant species on plots from South America, we used JUICE software [92], with the phi coefficient as a fidelity measure [94]. This coefficient is a standard method in phytosociological studies because the phi coefficient is independent of the number of plots in the dataset. JUICE standardizes all plot groups to an equal size, and we introduced the conditions of >30% of frequency percentage for each species, including a phi measure of >0.2. Species whose concentration in groups was not significant at *p* < 0.01 were disregarded [95].

The phytosociological names of the vegetation units are given according to the International Code of Phytosociological Nomenclature [96].

## Figures and Tables

**Table 1:** Similarity analysis of the montane rainforest per country according to the Sørensen coefficient.

	VEN	COLori	COLcen	COLocc	ECU	PERU	BOL	ARG
Plots; α Diversity	14; 226	39; 321	18; 821	39; 550	103; 819	106; 791	24; 334	21; 89
**VEN**	1	0.04753199	0.04011461	0.05412371	0.04784689	0.02359882	0.01403509	0
**COLori**	0.04753199	1	0.08581436	0.11710677	0.09649123	0.05215827	0.02406015	0.0097561
**COLcen**	0.04011461	0.08581436	1	0.11232677	0.11097561	0.05086849	0.01545064	0.01098901
**COLocc**	0.05412371	0.11710677	0.11232677	1	0.08473338	0.05965697	0.01342282	0.00625978
**ECU**	0.04784689	0.09649123	0.11097561	0.08473338	1	0.1378882	0.04127257	0.02863436
**PERU**	0.02359882	0.05215827	0.05086849	0.05965697	0.1378882	1	0.06696035	0.025
**BOL**	0.01403509	0.02406015	0.01545064	0.01342282	0.04127257	0.06696035	1	0.07852194
**ARG**	0	0.0097561	0.01098901	0.00625978	0.02863436	0.025	0.07852194	1

VEN: Venezuela, COLori: Eastern mountain range of Colombia, COLcen: Central mountain range of Colombia, COLocc: Western mountain range of Colombia, ECU: Ecuador, BOL: Bolivia, ARG: Argentina.

## Supplementary Materials

The following are available online at https://www.mdpi.com/2223-7747/9/12/1654/s1, Table S1: localities, bioclimatic belts, plant communities, and diagnostic plants from the rainforest studied in South America, Table S2: table with 364 plots studied in South America, Table S3: synthetic table of the plots studied in South America applying JUICE software.

## Appendix A

**Table A1:** Synthetic table of 106 plots carried out in montane rainforests of Peru.

Column Number	1	2	3	4	5	6	7	8	9	10	11	12	13	14	15	16	17	18	19	20	21	22	23	24
Columns of Table S3	71	72	73	74	75	76	77	78	79	80	81	82	83	84	85	86	87	88	89	90	91	92	93	94
Number of plots	1	5	7	7	3	6	1	5	3	5	4	7	2	2	8	5	2	4	3	6	5	1	4	10
Reference of plot intervals of Tables S2 and S3	214	215–219	220–226	227–233	234–236	237–242	243	244–248	249–251	252–256	257–260	261–267	268–269	270–271	272–279	280–284	285–286	287–290	291–293	294–299	300–304	305	306–309	310–319
Average altitude (m)	3786	2273	752	1440	2754	1713	575	2519	2719	2540	3277	3260	2559	150	2474	1818	2337	2400	2313	1355	2654	1762	865	124
***Mutisia cochabambensis-Polylepis incarum* community**																								
*Polylepis incarum* **(T)**	100	-	-	-	-	-	-	-	-	-	-	-	-	-	-	-	-	-	-	-	-	-	-	-
*Mutisia cochabambensis* **(C)**	100	-	-	-	-	-	-	-	-	-	-	-	-	-	-	-	-	-	-	-	-	-	-	-
*Citharexylum dentatum* **(S)**	100	-	-	-	-	-	-	-	-	-	-	-	-	-	-	-	-	-	-	-	-	-	-	-
*Elaphoglossum engelii* **(H)**	100	-	-	-	-	-	-	-	-	-	-	-	-	-	-	-	-	-	-	-	-	-	-	-
*Gaultheria vaccinioides* **(SS)**	100	-	-	-	-	-	-	-	-	-	-	-	-	-	-	-	-	-	-	-	-	-	-	-
***Smallantho parvicipitis-Oreopanacetum eriocephali* ass. nov.**																								
*Smallanthus parviceps* **(T)**	-	100	-	-	-	-	-	-	-	-	-	-	-	-	-	-	-	-	-	-	-	-	-	-
*Fuchsia boliviana* **(S)**	-	100	-	-	-	-	-	-	-	-	-	-	-	-	-	-	-	-	-	-	-	-	-	-
*Begonia bracteosa* **(H)**	-	100	-	14	-	-	-	-	-	-	-	-	-	-	-	-	-	-	-	-	-	-	-	-
*Melica scabra* **(H)**	-	80	-	-	-	-	-	-	-	-	-	-	-	-	-	-	-	-	-	-	-	-	-	-
*Miconia aff adinantha* **(S)**	-	100	-	29	-	-	-	-	-	-	-	-	-	-	25	-	-	-	-	-	-	-	-	-
*Phenax angustifolius* **(T)**	-	60	-	-	-	-	-	-	-	-	-	-	-	-	-	-	-	-	-	-	-	-	-	-
*Solanum aphyodendron* **(T)**	-	60	-	-	-	-	-	-	-	-	-	-	-	-	-	-	-	-	-	17	-	-	-	-
*Rubus boliviensis* **(S)**	-	60	-	-	-	-	-	-	-	-	-	-	-	-	-	-	-	-	-	-	-	-	-	-
*Pennisetum latifolium* **(H)**	-	40	-	-	-	-	-	-	-	-	-	-	-	-	-	-	-	-	-	-	-	-	-	-
*Croton regelianus* **(S)**	-	40	-	-	-	-	-	-	-	-	-	-	-	-	-	-	-	-	-	-	-	-	-	-
*Canna iridiflora* **(H)**	-	40	-	14	-	-	-	-	-	-	-	-	-	-	-	-	-	-	-	-	-	-	-	-
*Sambucus peruviana* **(T)**	-	40	-	-	-	-	-	-	-	-	-	-	-	-	-	-	-	-	-	-	-	-	-	-
***Iriartello setigerae-Cinchonetum micranthae* ass. nov.**																								
*Cinchona micrantha* **(T)**	-	-	100	-	-	-	-	-	-	-	-	-	-	-	-	-	-	-	-	-	-	-	-	-
*Iriartella setigera* **(S)**	-	-	100	-	-	-	-	-	-	-	-	-	-	-	-	-	-	-	-	-	-	-	-	-
*Cyathea subincisa* **(T)**	-	-	71	-	-	-	-	-	-	-	-	-	-	-	-	-	-	-	-	-	-	-	-	-
*Philodendron rudgeanum* **(Ep)**	-	-	71	-	-	-	-	-	-	-	-	-	-	-	-	-	-	-	-	-	-	-	-	-
*Heliconia hirsuta* **(H)**	-	-	86	-	-	-	-	-	-	-	-	-	-	-	-	-	-	-	-	-	-	-	25	-
*Ruagea insignis* **(T)**	-	-	57	-	-	-	-	-	-	-	-	-	-	-	-	-	-	-	-	-	-	-	-	-
*Dieffenbachia humilis* **(H)**	-	-	57	-	-	-	-	-	-	-	-	-	-	-	-	-	-	-	-	-	-	-	-	-
*Miconia lourteigiana* **(T)**	-	-	57	-	-	-	-	-	-	-	-	-	-	-	-	-	-	-	-	-	-	-	-	-
*Aphelandra aurantiaca* **(S)**	-	-	43	-	-	-	-	-	-	-	-	-	-	-	-	-	-	-	-	-	-	-	-	-
*Proteaceae_sg* **(T)**	-	-	43	-	-	-	-	-	-	-	-	-	-	-	-	-	-	-	-	-	-	-	-	-
*Smilax purhampuy* **(C)**	-	-	43	14	-	-	-	-	-	-	-	-	-	-	-	-	-	-	-	17	-	-	25	-
*Strychnos sp._sg* **(S)**	-	20	43	-	-	-	-	-	-	-	-	-	-	-	-	-	-	-	-	-	-	-	-	-
***Acalypho macrostachyae-Cecropietum polystachyae* ass. nov.**																								
*Cecropia polystachya* **(T)**	-	20	-	100	-	-	-	-	-	-	-	-	-	-	-	-	-	-	-	-	-	-	-	-
*Maranta arundinacea* **(H)**	-	-	-	43	-	-	-	-	-	-	-	-	-	-	-	-	-	-	-	-	-	-	-	-
*Acalypha macrostachya* **(T)**	-	-	-	43	-	-	-	-	-	-	-	-	-	-	-	-	-	-	-	17	-	-	25	-
***Crotono churumayensis-Hesperomeletum ferrugineae* ass. nov.**																								
*Croton churumayensis* **(T)**	-	-	-	-	100	-	-	-	-	-	-	-	-	-	-	-	-	-	-	-	-	-	-	-
*Cuphea cordata* **(S)**	-	-	-	-	100	-	-	-	-	-	-	-	-	-	-	-	-	-	-	-	-	-	-	-
*Tagetes elliptica* **(S)**	-	-	-	-	67	-	-	-	-	-	-	29	-	-	-	-	-	-	-	-	-	-	-	-
*Senna birostris* **(S)**	-	-	-	-	67	-	-	-	-	-	-	-	-	-	-	-	-	-	-	-	-	-	-	-
*Adiantum digitatum* **(H)**	-	-	-	-	67	-	-	-	-	-	-	-	-	-	-	-	-	-	-	-	-	-	-	-
*Peperomia sp._sg158* **(H)**	-	-	-	-	33	-	-	-	-	-	-	-	-	-	-	-	-	-	-	-	-	-	-	-
*Miconia alpina* **(S)**	-	-	-	-	33	-	-	-	-	-	-	-	-	-	-	-	-	-	-	-	-	-	-	-
*Gaultheria reticulata* **(S)**	-	-	-	-	33	-	-	-	-	-	-	-	-	-	-	-	-	-	-	-	-	-	-	-
*Ditassa sp._sg* **(Ep)**	-	20	-	-	33	-	-	-	-	-	-	-	-	-	-	-	-	-	-	-	-	-	-	-
*Baccharis genistelloides* **(SS)**	-	-	-	-	33	-	-	-	-	-	-	-	-	-	-	-	-	-	-	-	-	-	-	-
***Viburno reticulati-Weinmannietum spruceanae* ass. nov.**																								
*Viburnum reticulatum* **(T)**	-	-	-	-	-	83	-	-	-	-	-	-	-	-	-	-	-	-	-	-	-	-	-	-
*Pouzolzia poeppigiana* **(T)**	-	-	-	-	-	50	-	-	-	-	-	-	-	-	-	-	-	-	-	-	-	-	-	-
*Weinmannia spruceana* **(T)**	-	-	-	-	-	50	-	-	-	-	-	-	-	-	-	-	-	-	-	-	-	-	-	-
*Jatropha sp.* **(S)**	-	-	-	14	-	50	-	-	-	-	-	-	-	-	-	-	-	-	-	-	-	-	-	-
*Anthurium hamiltonii* **(H)**	-	-	-	-	-	33	-	-	-	-	-	-	-	-	-	-	-	-	-	-	-	-	-	-
*Mandevilla fragrans* **(C)**	-	-	-	-	-	33	-	-	-	-	-	-	-	-	-	-	-	-	-	-	-	-	-	-
*Clusia crenata* **(T)**	-	-	-	-	-	33	-	-	-	-	-	-	-	-	-	-	-	-	-	-	-	-	-	-
*Pitcairnia paniculata* **(H)**	-	-	-	-	-	33	-	-	-	-	-	-	-	-	-	-	-	-	-	-	-	-	-	-
*Rubus urticifolius* **(S)**	-	-	-	14	-	33	-	-	-	-	-	-	-	-	-	-	-	-	-	-	-	-	-	-
*Gleichenella pectinata* **(H)**	-	-	-	-	-	33	-	-	-	-	-	-	-	-	-	-	-	-	-	-	-	-	-	-
***Muntingia calabura-Hura crepitans* community**																								
*Hura crepitans* **(T)**	-	-	-	-	-	-	100	-	-	-	-	-	-	-	-	-	-	-	-	-	-	-	-	-
*Ruellia brevifolia* **(S)**	-	-	-	-	-	-	100	-	-	-	-	-	-	-	-	-	-	-	-	-	-	-	-	-
*Albizia multiflora* **(T)**	-	-	-	-	-	-	100	-	-	-	-	-	-	-	-	-	-	-	-	-	-	-	-	-
*Piper peltatum* **(H)**	-	-	-	-	-	-	100	-	-	-	-	-	-	-	-	-	-	-	-	-	-	-	-	-
*Piper nudilimbum* **(S)**	-	-	-	-	-	-	100	-	-	-	-	-	-	-	-	-	-	-	-	-	-	-	-	-
*Ichnanthus nemorosus* **(H)**	-	-	-	-	-	-	100	-	-	-	-	-	-	-	-	-	-	-	-	-	-	-	-	-
*Leucaena trichodes* **(T)**	-	-	-	-	-	-	100	-	-	-	-	-	-	-	-	-	-	-	-	-	-	-	-	-
*Muntingia calabura* **(T)**	-	-	-	-	-	-	100	-	-	-	-	-	-	-	-	-	-	-	-	-	-	-	-	-
*Paullinia alata* **(C)**	-	-	29	14	-	17	100	-	-	-	-	-	-	-	-	-	-	-	-	-	-	100	-	-
***Axinaeo nitidae-Podocarpetum oleifolii***																								
*Axinaea nitida* **(S)**	-	-	-	-	-	-	-	100	33	-	-	-	-	-	38	40	-	-	-	-	-	-	-	-
*Podocarpus oleifolius* **(T)**	-	-	-	-	-	-	-	100	20	-	-	-	-	-	-	-	-	-	-	-	-	-	-	-
*Trichilia tomentosa* **(T)**	-	-	-	-	-	-	-	100	-	-	-	-	-	-	-	-	-	-	-	-	-	-	-	-
*Chrysophyllum* *contumazense* **(T)**	-	-	-	-	-	-	-	60	-	-	-	-	-	-	-	-	-	-	-	-	-	-	-	-
*Miconia firma* **(S)**	-	-	-	-	-	-	-	40	-	-	-	-	-	-	-	-	-	-	-	-	-	-	-	-
*Viola argute* **(C)**	-	-	-	-	-	-	-	40	-	-	-	-	-	-	-	-	-	-	-	-	-	-	-	-
***Verbesino auriculigerae-Siparunetum muricatae***																								
*Siparuna muricata* **(T)**	-	-	-	-	-	-	-	40	100	100	-	-	-	-	25	-	-	-	33	50	-	-	-	-
*Verbesina auriculigera* **(S)**	-	-	-	-	-	-	-	-	100	40	25	-	100	-	-	-	-	-	-	-	-	-	-	-
*Cervantesia tomentosa* **(T)**	-	-	-	-	-	-	-	-	67	-	-	-	-	-	-	-	-	-	-	-	-	-	-	-
*Meliosma frondosa* **(T)**	-	-	-	-	-	-	-	-	33	-	-	-	-	-	-	-	-	-	-	-	-	-	-	-
*Valeriana asterothrix* **(H)**	-	-	-	-	-	-	-	-	33	20	25	-	-	-	-	-	-	-	-	-	-	-	-	-
*Solanum caripense* **(C)**	-	-	-	-	-	-	-	-	33	20	-	-	-	-	-	-	-	-	-	-	-	-	-	-
*Calceolaria calycina* **(S)**	-	-	-	-	-	-	-	-	33	-	-	-	-	-	-	-	-	-	-	-	-	-	-	-
*Piper barbatum* **(C)**	-	-	-	-	-	-	-	-	33	-	-	29	-	-	13	-	-	-	-	-	-	-	-	-
*Berberis buceronis* **(S)**	-	-	-	-	-	-	-	-	33	-	-	-	-	-	-	-	-	-	-	-	-	-	-	-
*Munnozia ferreyrii* **(H)**	-	-	-	-	-	-	-	-	67	-	-	-	-	-	-	-	-	-	-	-	-	-	-	-
*Pappobolus sp._cjvs8* **(S)**	-	-	-	-	-	-	-	-	33	-	-	-	-	-	-	-	-	-	-	-	-	-	-	-
*Phenax laxiflorus* **(S)**	-	-	-	-	-	-	-	-	33	-	-	-	-	-	-	-	-	-	-	-	-	-	-	-
*Lepechinia lamiifolia* **(S)**	-	-	-	-	-	-	-	-	33	-	-	-	-	-	-	-	-	-	-	-	-	-	-	-
*Scutia spicata* **(S)**	-	-	-	-	-	-	-	-	33	-	-	-	-	-	-	-	-	-	-	-	-	-	-	-
*Siphocampylus* *macropodoides* **(S)**	-	-	-	-	-	-	-	-	33	-	-	-	-	-	-	-	-	-	-	-	-	-	-	-
*Wedelia latifolia* **(S)**	-	-	-	-	-	-	-	-	33	-	-	-	-	-	-	-	-	-	-	-	-	-	-	-
*Symplocos sandemanii* **(S)**	-	-	-	-	-	-	-	-	33	-	-	-	-	-	-	-	-	-	-	-	-	-	-	-
*Fabaceae_cjvs6* **(T)**	-	-	-	-	-	-	-	-	33	-	-	-	-	-	-	-	-	-	-	-	-	-	-	-
*Begonia acerifolia* **(H)**	-	-	-	-	-	-	-	-	33	-	-	-	-	-	-	-	-	-	-	-	-	-	-	-
*Rubus sp._cjvs6* **(S)**	-	-	-	-	-	-	-	-	33	-	-	-	-	-	-	-	-	-	-	-	-	-	-	-
*Luffa operculata* **(C)**	-	-	-	-	-	-	-	-	33	-	25	-	-	-	-	-	-	-	-	-	-	-	-	-
*Mikania sp._cjvs6* **(C)**	-	-	-	-	-	-	-	-	33	-	-	-	-	-	-	-	-	-	-	-	-	-	-	-
*Paspalum candidum* **(H)**	-	-	-	-	-	-	-	-	33	-	25	-	-	-	-	-	-	-	-	-	-	-	-	-
***Berberido beauverdianae-Myrcianthetum myrsinoidis***																								
*Maytenus boarioides* **(S)**	-	-	-	-	-	-	-	-	-	80	-	-	-	-	-	-	-	-	-	-	-	-	-	-
*Viburnum mathewsii* **(T)**	-	-	-	-	-	-	-	-	-	60	-	-	-	-	-	-	-	-	-	-	-	-	-	-
*Bidens squarrosa* **(H)**	-	-	-	-	-	-	-	-	-	40	-	-	-	-	-	-	-	-	-	-	-	-	-	-
*Bomarea cornuta* **(C)**	-	-	-	-	-	-	-	-	-	40	-	-	-	-	-	-	-	-	-	-	20	-	-	-
*Tecoma stans* **(T)**	-	-	-	-	-	-	-	20	-	40	-	-	-	-	-	-	-	-	-	-	-	-	-	-
*Salvia oppositiflora* **(S)**	-	-	-	-	-	-	-	-	-	40	25	-	-	-	-	-	-	-	-	-	-	-	-	-
*Liabum solidagineum* **(S)**	-	-	-	-	-	-	-	20	-	60	-	-	-	-	-	-	-	-	-	-	-	-	-	-
*Thalictrum longistylum* **(H)**	-	20	-	-	-	-	-	-	-	40	-	-	-	-	-	-	-	-	-	-	-	-	-	-
***Aristeguietio discoloris-Kageneckietum lanceolatae***																								
*Kageneckia lanceolata* **(T)**	-	-	-	-	-	-	-	-	-	-	100	-	-	-	-	-	-	-	-	-	-	-	-	-
*Aristeguietia discolor* **(S)**	-	-	-	-	-	-	-	-	-	-	100	-	-	-	-	-	-	-	-	-	-	-	-	-
*Baccharis emarginata* **(S)**	-	-	-	-	-	-	-	-	-	-	100	-	-	-	-	-	-	-	-	-	-	-	-	-
*Calceolaria rugulosa* **(S)**	-	-	-	-	-	-	-	-	-	-	100	-	-	-	-	-	-	-	-	-	-	-	-	-
*Colletia spinosissima* **(S)**	-	-	-	-	-	-	-	-	-	-	100	-	-	-	-	-	-	-	-	-	-	-	-	-
*Cronquistianthus urubambensis* **(S)**	-	-	-	14	-	-	-	-	-	-	100	-	-	-	-	-	-	-	-	-	-	-	-	-
*Coreopsis fasciculate* **(SS)**	-	-	-	-	-	-	-	-	-	-	75	-	-	-	-	-	-	-	-	-	-	-	-	-
*Scutellaria scutellarioides* **(SS)**	-	-	-	-	-	-	-	-	-	-	50	-	-	-	-	-	-	-	-	-	-	-	-	-
*Passiflora peduncularis* **(C)**	-	-	-	-	-	-	-	-	-	-	50	-	-	-	-	-	-	-	-	-	-	-	-	-
*Eryngium weberbaueri* **(H)**	-	-	-	-	-	-	-	-	-	-	50	-	-	-	-	-	-	-	-	-	-	-	-	-
***Baccharito latifoliae-Monactinetum flaverioidis***																								
*Passiflora tarminiana* **(C)**	-	-	-	-	-	-	-	-	-	-	-	57	-	-	-	-	-	-	-	-	-	-	-	-
*Salvia alborosea* **(S)**	-	-	-	-	-	-	-	-	-	-	-	57	-	-	-	20	-	-	-	-	-	-	-	-
*Cronquistianthus* *glomeratus* **(S)**	-	-	-	-	-	-	-	-	-	-	-	43	-	-	-	-	-	-	-	-	-	-	-	-
*Calceolaria bicrenata* **(S)**	-	-	-	-	-	-	-	-	-	-	-	43	-	-	-	-	-	-	-	-	-	-	-	-
*Minthostachys mollis* **(S)**	-	20	-	-	-	-	-	-	-	-	-	43	-	-	-	-	-	-	-	-	-	-	-	-
***Monactino flaverioidis-Colignonietum parviflorae***																								
*Colignonia parviflora* **(S)**	-	-	-	-	-	-	-	-	-	-	-	-	100	-	-	-	-	-	-	-	-	-	-	-
*Alternanthera villosa* **(H)**	-	-	-	-	-	-	-	-	-	20	-	-	100	-	-	-	-	-	-	-	-	-	-	-
*Heliotropium incanum* **(H)**	-	-	-	-	-	-	-	-	-	-	-	-	50	-	-	-	-	-	-	-	-	-	-	-
*Urtica peruviana* **(H)**	-	-	-	-	-	-	-	20	-	-	-	-	50	-	-	-	-	-	-	-	-	-	-	-
*Viguiera procumbens* **(S)**	-	-	-	-	-	-	-	-	-	-	25	14	50	-	-	-	-	-	-	-	-	-	-	-
***Oenocarpo maporae-Mauritietum flexuosae***																								
*Oenocarpus mapora* **(T)**	-	-	-	-	-	-	-	-	-	-	-	-	-	100	-	-	-	-	-	-	-	-	-	-
*Mauritia flexuosa* **(T)**	-	-	-	-	-	-	-	-	-	-	-	-	-	100	-	-	-	-	-	-	-	-	-	-
*Buchenavia oxycarpa* **(T)**	-	-	-	-	-	-	-	-	-	-	-	-	-	100	-	-	-	-	-	-	-	-	-	-
*Ficus americana subsp. guianensis* **(T)**	-	-	-	-	-	-	-	-	-	-	-	-	-	100	-	-	-	-	-	-	-	-	-	-
*Dialium guianense* **(T)**	-	-	-	-	-	-	-	-	-	-	-	-	-	100	-	-	-	-	-	-	-	-	-	-
*Virola decorticans* **(T)**	-	-	-	-	-	-	-	-	-	-	-	-	-	100	-	-	-	-	-	-	-	-	-	-
*Symphonia globulifera* **(T)**	-	-	-	-	-	-	-	-	-	-	-	-	-	100	-	-	-	-	-	-	-	-	-	-
*Geonoma macrostachys* **(S)**	-	-	-	-	-	-	-	-	-	-	-	-	-	100	-	-	-	-	-	-	-	-	-	-
*Rinorea racemosa* **(S)**	-	-	-	-	-	-	-	-	-	-	-	-	-	100	-	-	-	-	-	-	-	-	-	-
*Agonandra sylvatica* **(T)**	-	-	-	-	-	-	-	-	-	-	-	-	-	100	-	-	-	-	-	-	-	-	-	-
*Planchonella obovata* **(T)**	-	-	-	-	-	-	-	-	-	-	-	-	-	100	-	-	-	-	-	-	-	-	-	-
*Duguetia quitarensis* **(T)**	-	-	-	-	-	-	-	-	-	-	-	-	-	100	-	-	-	-	-	-	-	-	-	-
*Bactris concinna* **(S)**	-	-	-	-	-	-	-	-	-	-	-	-	-	100	-	-	-	-	-	-	-	-	-	-
*Inga sp. 01_jhom11* **(T)**	-	-	-	-	-	-	-	-	-	-	-	-	-	50	-	-	-	-	-	-	-	-	-	-
*Costus scaber* **(H)**	-	-	-	-	-	-	-	-	-	-	-	-	-	50	-	-	-	-	-	-	-	-	-	-
*Virola calophylla* **(S)**	-	-	-	-	-	-	-	-	-	-	-	-	-	50	-	-	-	-	-	-	-	-	-	-
*Ceiba pentandra* **(T)**	-	-	-	-	-	-	-	-	-	-	-	-	-	50	-	-	-	-	-	-	-	-	-	-
*Anthurium apaporanum* **(H)**	-	-	-	-	-	-	-	-	-	-	-	-	-	50	-	-	-	-	-	-	-	-	-	-
*Terminalia amazonia* **(T)**	-	-	-	-	-	-	-	-	-	-	-	-	-	50	-	-	-	-	-	-	-	-	-	-
*Chrysobalanaceae_jhom11* **(T)**	-	-	-	-	-	-	-	-	-	-	-	-	-	50	-	-	-	-	-	-	-	-	-	-
*Euterpe precatoria* **(T)**	-	-	-	-	-	-	-	-	-	-	-	-	-	50	-	-	-	-	-	-	-	-	-	-
*Hevea guianensis* **(T)**	-	-	-	-	-	-	-	-	-	-	-	-	-	50	-	-	-	-	-	-	-	-	-	-
*Socratea exorrhiza* **(T)**	-	-	-	-	-	-	-	-	-	-	-	-	-	50	-	-	-	-	-	-	-	-	-	-
***Hypolepido parallelogrammae-Alnetum acuminatae* ass. nov.**																								
*Cyathea herzogii* **(T)**	-	-	-	-	-	-	-	-	-	-	-	-	-	-	50	-	-	-	-	-	-	-	-	-
*Hypolepis* *parallelogramma* **(H)**	-	-	-	-	-	17	-	-	-	-	-	-	-	-	63	-	-	-	-	-	-	-	-	-
*Hypolepis obtusata* **(H)**	-	-	-	-	-	-	-	-	-	-	-	-	-	-	50	-	-	-	-	-	-	-	-	-
*Aulonemia longiaristata* **(H)**	-	-	-	-	-	-	-	-	-	-	-	-	-	-	38	-	-	-	-	-	-	-	-	-
*Miconia aff asperrima* **(S)**	-	-	-	-	-	-	-	-	-	-	-	-	-	-	38	-	-	-	-	-	-	-	-	-
*Gurania sp. 01_cut* **(T)**	-	-	-	-	-	-	-	-	-	-	-	-	-	-	38	-	-	-	-	-	-	-	-	-
*Solanum sp. 02_cut* **(S)**	-	-	-	-	-	-	-	-	-	-	-	-	-	-	38	-	-	-	-	-	-	-	-	-
*Acanthus ilicifolius* **(S)**	-	-	-	-	-	-	-	-	-	-	-	-	-	-	38	-	-	-	-	-	-	-	-	-
*Inga acuminata* **(T)**	-	-	-	-	-	-	-	-	-	-	-	-	-	-	38	-	-	-	-	-	-	-	-	-
*Miconia sp. 03_cut* **(S)**	-	-	-	-	-	-	-	-	-	-	-	-	-	-	38	-	-	-	-	-	-	-	-	-
*Hedyosmum sprucei* **(S)**	-	-	-	-	-	-	-	-	-	-	-	-	-	-	38	-	-	-	-	-	-	-	-	-
*Miconia aggregata* **(S)**	-	-	-	-	-	-	-	-	-	-	-	-	-	-	38	-	-	-	-	-	-	-	-	-
*Asplenium auriculatum* **(H)**	-	-	-	-	-	-	-	-	-	-	-	14	-	-	38	-	-	-	-	-	-	-	-	-
*Cyathea lechleri* **(T)**	-	-	-	-	-	-	-	-	-	-	-	-	-	-	38	-	-	-	-	-	20	-	-	-
*Geonoma orbignyana* **(S)**	-	-	-	-	-	-	-	-	-	-	-	-	-	-	38	-	-	-	-	-	-	-	-	-
*Saccoloma inaequale* **(H)**	-	-	-	-	-	-	-	-	-	-	-	-	-	-	38	-	-	-	-	-	-	-	-	-
*Serpocaulon fraxinifolium* **(H)**	-	-	-	-	-	-	-	-	-	-	-	-	-	-	38	20	-	-	-	-	-	-	-	-
***Lueheo divaricatae-Ceroxylonetum peruviani* ass. nov.**																								
*Luehea divaricata* **(T)**	-	-	-	-	-	-	-	-	-	-	-	-	-	-	-	100	-	-	-	-	-	-	-	-
*Ceroxylon peruvianum* **(T)**	-	-	-	-	-	-	-	-	-	-	-	-	-	-	-	100	-	-	-	-	-	-	-	-
*Styloceras laurifolium* **(S)**	-	-	-	-	-	-	-	-	-	-	-	-	-	-	-	80	-	-	-	-	-	-	-	-
*Pueraria phaseoloides* **(C)**	-	-	-	-	-	-	-	-	-	-	-	-	-	-	-	60	-	-	-	-	-	-	-	-
*Solanum sp. 03* **(S)**	-	-	-	-	-	17	-	-	-	-	-	14	-	-	13	60	-	-	-	-	-	-	-	-
*Austroeupatorium inulaefolium* **(S)**	-	-	-	-	-	-	-	-	-	-	-	-	-	-	-	40	-	-	-	-	-	-	-	-
*Schistocarpha sinforosi* **(S)**	-	-	-	-	-	-	-	-	-	-	-	-	-	-	13	40	-	-	-	-	-	-	-	-
*Viburnum incarum* **(S)**	-	-	-	-	-	-	-	-	-	-	-	-	-	-	13	40	-	-	-	-	-	-	-	-
*Ladenbergia oblongifolia* **(T)**	-	-	-	-	-	-	-	-	-	-	-	-	-	-	-	40	-	-	-	17	-	-	25	-
***Mutisio wurdackii-Alnetum acuminatae* ass. nov.**																								
*Mutisia wurdackii* **(C)**	-	-	-	-	-	-	-	-	-	-	-	-	-	-	-	-	100	25	-	-	-	-	-	-
*Weinmannia costulata* **(S)**	-	-	-	-	-	-	-	-	-	-	-	-	-	-	-	-	100	-	-	-	-	-	-	-
*Cortaderia jubata* **(H)**	-	-	-	-	-	-	-	-	-	-	-	-	-	-	-	-	50	-	-	-	-	-	-	-
***Axinaeo tomentosae-Ceroxylonetum quindiuensis* ass. nov.**																								
*Ceroxylon quindiuense* **(T)**	-	-	-	-	-	-	-	-	-	-	-	-	-	-	-	-	-	100	-	-	-	-	-	-
*Cedrela odorata* **(T)**	-	-	-	-	-	-	-	-	-	-	-	-	-	-	25	-	-	100	-	-	-	-	-	-
*Axinaea tomentosa* **(S)**	-	-	-	-	-	-	-	-	-	-	-	-	-	-	-	-	-	75	-	-	-	-	-	-
*Cronquistianthus chachapoyensis* **(S)**	-	-	-	-	-	-	-	-	-	-	-	-	-	-	13	20	-	75	-	-	-	-	-	-
*Citronella incarum* **(T)**	-	-	-	-	-	-	-	-	-	-	-	-	-	-	-	-	-	50	-	-	-	-	-	-
*Vismia pozuzoensis* **(T)**	-	-	-	-	-	-	-	-	-	-	-	-	-	-	-	-	-	50	-	-	-	-	-	-
*Erythrina aff edulis* **(T)**	-	-	-	-	-	-	-	-	-	-	-	-	-	-	-	-	-	50	-	-	-	-	-	-
*Solanum barbulatum* **(S)**	-	-	-	-	-	-	-	-	-	-	-	-	-	-	-	-	-	50	-	-	-	-	-	-
*Hedyosmum lechleri* **(T)**	-	-	-	-	-	-	-	-	-	-	-	-	-	-	-	-	-	50	-	-	20	-	-	-
*Viburnum pichinchense* **(T)**	-	-	-	-	-	-	-	-	-	-	-	-	-	-	-	-	-	50	-	-	-	-	-	-
*Cornus peruviana* **(S)**	-	-	-	-	-	-	-	-	-	-	-	-	-	-	-	-	-	50	-	-	-	-	-	-
***Piperi chanchamayani-Cecropietum strigosae* ass. nov.**																								
*Pennisetum* *aff peruvianum* **(H)**	-	-	-	-	-	-	-	-	-	-	-	-	-	-	-	-	-	-	100	-	-	-	-	-
*Ageratina tambillensis* **(S)**	-	-	-	-	-	-	-	-	-	-	-	-	-	-	-	-	-	-	100	17	20	-	-	-
*Solanum mite* **(S)**	-	-	-	-	-	-	-	-	-	-	-	-	-	-	-	-	-	-	67	-	-	-	-	-
*Centropogon altus* **(S)**	-	-	-	-	-	-	-	-	-	-	-	-	-	-	-	-	-	-	67	-	-	-	-	-
*Cecropia angustifolia* **(T)**	-	-	-	-	-	-	-	-	-	-	-	-	-	-	25	-	-	-	67	17	-	-	-	-
*Cecropia strigose* **(T)**	-	-	-	-	-	-	-	-	-	-	-	-	-	-	-	-	-	-	67	-	-	-	-	-
*Clusia aff pallida* **(T)**	-	-	-	-	-	-	-	-	-	-	-	-	-	-	-	-	-	-	67	-	-	-	-	-
*Cavendishia punctate* **(S)**	-	-	-	-	-	-	-	-	-	-	-	-	-	-	-	-	-	-	33	-	-	-	-	-
*Pilea aff bassleriana* **(S)**	-	-	-	-	-	-	-	-	-	-	-	-	-	-	-	-	-	-	33	-	-	-	-	-
*Pilea pavonii* **(S)**	-	-	-	-	-	-	-	-	-	-	-	-	-	-	-	-	-	-	33	-	-	-	-	-
*Palicourea sp nov?sat2* **(S)**	-	-	-	-	-	-	-	-	-	-	-	-	-	-	-	-	-	-	33	-	-	-	-	-
*Acinodendron* *aff ferrugineum* **(S)**	-	-	-	-	-	-	-	-	-	-	-	-	-	-	-	-	-	-	33	-	-	-	-	-
*Thelypteris euchlora* **(H)**	-	-	-	-	-	-	-	-	-	-	-	-	-	-	-	-	-	-	33	-	-	-	-	-
*Adianthum raddianum* **(H)**	-	-	-	-	-	-	-	-	-	-	-	-	-	-	-	-	-	-	33	-	-	-	-	-
*Coriaria myrtifolia* **(S)**	-	-	-	-	-	-	-	-	-	-	-	-	-	-	-	-	-	-	33	-	-	-	-	-
*Solanum asperolanatum* **(S)**	-	-	-	-	-	-	-	-	-	-	-	-	-	-	-	-	-	25	33	-	-	-	-	-
*Miconia sanguinea* **(S)**	-	-	-	-	-	-	-	-	-	-	-	-	-	-	-	-	-	-	33	17	-	-	-	-
*Canna paniculata* **(H)**	-	-	-	-	-	-	-	-	-	-	-	-	-	-	-	-	-	-	33	17	-	-	-	-
*Chromolaena leptocephala* **(S)**	-	-	-	-	-	-	-	-	-	-	-	-	-	-	-	-	-	-	33	-	20	-	-	-
*Miconia sp. 08_sat* **(S)**	-	-	-	-	-	-	-	-	-	-	-	-	-	-	-	-	-	-	33	17	-	-	-	-
*Meriania tomentosa* **(S)**	-	-	-	-	-	-	-	-	-	-	-	-	-	-	-	-	-	-	33	20	-	-	-	-
*Psammisia coarctata* **(S)**	-	-	-	-	-	-	-	-	-	-	-	-	-	-	-	-	-	-	33	-	-	-	-	-
*Anthurium grande* **(H)**	-	-	-	29	-	17	-	-	-	-	-	-	-	-	-	-	-	-	33	-	-	-	-	-
***Cedrelo fissilis-Ficetum maximae* ass. nov.**																								
*Ficus maxima* **(T)**	-	-	-	-	-	-	-	-	-	-	-	-	-	-	-	-	-	-	-	100	-	-	-	-
*Dieffenbachia sp. 02_sat* **(H)**	-	-	-	-	-	-	-	-	-	-	-	-	-	-	-	-	-	-	-	83	-	-	-	-
*Dioscorea sp. 01_sat* **(C)**	-	-	-	-	-	-	-	-	-	-	-	-	-	-	-	-	-	-	-	67	-	-	-	-
*Inga adenophylla* **(T)**	-	-	-	-	-	-	-	-	-	-	-	-	-	-	-	-	-	-	-	67	-	-	-	-
*Serjania communis* **(C)**	-	-	-	-	-	-	-	-	-	-	-	-	-	-	-	-	-	-	-	67	-	-	-	-
*Begonia parviflora* **(C)**	-	-	-	-	-	-	-	-	-	-	-	-	-	-	25	-	-	-	-	67	20	-	-	-
*Heliconia subulata* **(H)**	-	-	-	-	-	-	-	-	-	-	-	-	-	-	-	20	-	-	-	67	-	-	-	-
*Lastreopsis effuse* **(H)**	-	-	-	-	-	-	-	-	-	-	-	-	-	-	-	-	-	-	-	67	-	-	-	-
*Ocotea obovata* **(T)**	-	-	-	-	-	-	-	-	-	-	-	-	-	-	-	-	-	-	-	50	-	-	-	-
*Centropogon granulosus* **(C)**	-	-	-	-	-	-	-	-	-	-	-	-	-	-	-	-	-	-	-	50	-	-	-	-
*Philodendron aff killipii* **(Ep)**	-	-	-	-	-	-	-	-	-	-	-	-	-	-	-	-	-	-	-	50	-	-	-	-
*Selaginella trisulcata* **(H)**	-	-	-	-	-	-	-	-	-	-	-	-	-	-	-	-	-	-	-	50	-	-	-	-
*Croton lechheri* **(T)**	-	-	-	-	-	-	-	-	-	-	-	-	-	-	-	-	-	-	-	50	-	-	-	-
*Campyloneurum* *ophicaulon* **(H)**	-	-	-	-	-	-	-	-	-	-	-	-	-	-	-	-	-	-	-	50	-	-	-	-
*Prestoea carderi* **(S)**	-	-	-	-	-	-	-	-	-	-	-	-	-	-	-	-	-	-	-	50	20	-	-	-
*Vernonanthura patens* **(S)**	-	-	-	-	-	-	-	-	-	-	-	-	-	-	-	20	-	-	-	50	-	-	-	-
*Urera baccifera* **(S)**	-	-	-	-	-	-	-	-	-	-	-	-	-	-	25	-	-	-	-	50	-	-	25	-
*Alsophila mostellaria* **(T)**	-	-	-	-	-	-	-	-	-	-	-	-	-	-	-	-	-	-	-	33	-	-	-	-
*Thelypteris jamesonii* **(H)**	-	-	-	-	-	-	-	-	-	-	-	-	-	-	-	-	-	-	-	33	-	-	-	-
*Melastomataceae 02_sat* **(S)**	-	-	-	-	-	-	-	-	-	-	-	-	-	-	-	-	-	-	-	33	-	-	-	-
*Thelypteris salzmannii* **(H)**	-	-	-	-	-	-	-	-	-	-	-	-	-	-	-	-	-	-	-	33	-	-	-	-
*Costus sp. 02_sat* **(H)**	-	-	-	-	-	-	-	-	-	-	-	-	-	-	-	-	-	-	-	33	-	-	-	-
*Urera verrucosa* **(S)**	-	-	-	-	-	-	-	-	-	-	-	-	-	-	-	-	-	-	-	33	-	-	-	-
*Columnea inaquilatera* **(H)**	-	-	-	-	-	-	-	-	-	-	-	-	-	-	-	-	-	-	-	33	-	-	-	-
*Serpocaulon loriceum* **(H)**	-	-	-	-	-	-	-	-	-	-	-	-	-	-	25	-	-	25	-	33	-	-	-	-
*Pteris altissima* **(H)**	-	-	-	-	-	-	-	-	-	-	-	-	-	-	-	-	-	-	-	33	-	-	-	-
*Nephrolepis pectinata* **(H)**	-	-	-	-	-	-	-	-	-	-	-	-	-	-	-	-	-	-	-	33	-	-	-	-
*Bignoniaceae* **(C)**	-	-	-	14	-	-	-	-	-	-	-	-	-	-	-	20	-	-	-	33	-	-	-	-
***Cecropio albicantis-Weinmannietum glomeratae* ass. nov.**																								
*Cecropia albicans* **(T)**	-	-	-	-	-	-	-	-	-	-	-	-	-	-	-	-	-	-	-	-	60	-	-	-
*Weinmannia latifolia* **(T)**	-	-	-	-	-	-	-	-	-	-	-	-	-	-	13	-	-	-	-	-	60	-	-	-
*Solanum aff sycophanta* **(S)**	-	-	-	-	-	-	-	-	-	-	-	-	-	-	-	-	-	-	-	-	60	-	-	-
*Tibouchina saxosa* **(SS)**	-	-	-	-	-	-	-	-	-	-	-	-	-	-	-	-	-	-	-	-	60	-	-	-
*Sticherus revolutus* **(H)**	-	-	-	-	-	-	-	-	-	-	-	-	-	-	-	-	-	-	-	-	60	-	-	-
*Cavendishia nolbilis* **(S)**	-	-	-	-	-	-	-	-	-	-	-	-	-	-	-	-	-	-	-	-	60	-	-	-
*Piper augustum* **(S)**	-	-	-	-	-	-	-	-	-	-	-	-	-	-	-	-	-	-	-	-	40	-	25	-
*Oreopanax* *aff cheirophyllus* **(T)**	-	-	-	-	-	-	-	-	-	-	-	-	-	-	-	-	-	-	-	-	40	-	-	-
*Vaccinium crenatum* **(SS)**	-	-	-	-	-	-	-	-	-	-	-	-	-	-	-	-	-	-	-	-	40	-	-	-
*Miconia galactantha* **(S)**	-	-	-	-	-	-	-	-	-	-	-	-	-	-	-	-	-	-	-	-	40	-	-	-
*Centropogon reflexus* **(C)**	-	-	-	-	-	-	-	-	-	-	-	-	-	-	-	-	-	-	-	-	40	-	-	-
*Dioscorea mitoensis* **(C)**	-	-	-	-	-	-	-	-	-	-	-	-	-	-	-	-	-	-	-	-	40	-	-	-
*Weinmannia auriculata* **(T)**	-	-	-	-	-	-	-	-	-	-	-	-	-	-	-	-	-	-	-	-	40	-	-	-
*Centropogon hirtus* **(S)**	-	-	-	-	-	-	-	-	-	-	-	-	-	-	-	-	-	-	-	-	40	-	-	-
*Ageratina rhytidodes* **(S)**	-	-	-	-	-	-	-	-	-	-	-	-	-	-	-	-	-	-	-	-	40	-	-	-
*Thelypteris pachyrhachys* **(H)**	-	-	-	-	-	-	-	-	-	-	-	-	-	-	-	-	-	-	-	-	40	-	-	-
*Bomarea pseudopurpurea* **(C)**	-	-	-	-	-	-	-	-	-	-	-	-	-	-	-	-	-	-	-	-	40	-	-	-
*Elleanthus aurantiacus* **(H)**	-	-	-	-	-	-	-	-	-	-	-	-	-	-	-	-	-	-	-	-	40	-	-	-
*Weinmannia microphylla* **(S)**	-	-	-	-	-	-	-	-	-	-	-	-	-	-	-	-	-	-	-	-	40	-	-	-
*Tibouchina lepidota* **(SS)**	-	-	-	-	-	-	-	-	-	-	-	-	-	-	-	-	-	-	-	-	40	-	-	-
*Disterigma acuminatum* **(SS)**	-	-	-	-	-	-	-	-	-	-	-	-	-	-	-	-	-	-	-	-	40	-	-	-
***Clusia trochiformis-Cecropia utcubambana* community**																								
*Fuchsia macrophylla* **(S)**	-	-	-	-	-	-	-	-	-	-	-	-	-	-	-	-	-	-	-	-	20	100	-	-
*Serpocaulon adnatum* **(H)**	-	-	-	-	-	-	-	-	-	-	-	-	-	-	-	-	-	-	-	-	20	100	25	-
*Barnadesia corymbosa* **(S)**	-	-	-	-	-	-	-	-	-	-	-	-	-	-	20	-	-	-	-	-	-	100	-	-
*Ipomoea sp._tin_3* **(C)**	-	-	-	-	-	-	-	-	-	-	-	-	-	-	-	-	-	-	-	-	-	100	-	-
*Anthurium sp._tin_3* **(H)**	-	-	-	-	-	-	-	-	-	-	-	-	-	-	-	-	-	-	-	-	-	100	-	-
*Piper semicordulatum* **(T)**	-	-	-	-	-	-	-	-	-	-	-	-	-	-	-	-	-	-	-	-	-	100	-	-
*Canna bangii* **(H)**	-	-	-	-	-	-	-	-	-	-	-	-	-	-	-	-	-	-	-	-	-	100	-	-
*Margaritopsis boliviana* **(S)**	-	-	-	-	-	-	-	-	-	-	-	-	-	-	-	-	-	-	-	-	-	100	-	-
*Schefflera pentandra* **(T)**	-	-	-	-	-	-	-	-	-	-	-	-	-	-	-	-	-	-	-	-	-	100	-	-
*Dryopteris patula* **(H)**	-	-	-	-	-	-	-	-	-	-	-	-	-	-	-	-	-	-	-	-	-	100	25	-
*Palicourea angustifolia* **(S)**	-	-	-	-	-	-	-	-	-	-	-	-	-	-	-	-	-	-	-	-	-	100	-	-
***Chelyocarpo ulei-Acacietum loretensis* ass. nov.**																								
*Acacia loretensis* **(T)**	-	-	-	-	-	-	-	-	-	-	-	-	-	-	-	-	-	-	-	-	-	-	100	-
*Chelyocarpus ulei* **(S)**	-	-	-	-	-	-	-	-	-	-	-	-	-	-	-	-	-	-	-	-	-	-	100	-
*Miconia aff cremophylla* **(S)**	-	-	-	-	-	-	-	-	-	-	-	-	-	-	-	-	-	-	-	-	-	-	100	-
*Selaginella haematodes* **(H)**	-	-	-	-	-	-	-	-	-	-	-	-	-	-	-	-	-	-	-	-	-	-	75	-
*Acalypha stricta* **(S)**	-	-	-	-	-	-	-	-	-	-	-	-	-	-	-	-	-	-	-	-	-	-	75	-
*Banara guianensis* **(T)**	-	-	-	-	-	-	-	-	-	-	-	-	-	-	-	-	-	-	-	-	20	-	50	-
*Heliconia rostrate* **(H)**	-	-	-	-	-	-	-	-	-	-	-	-	-	-	-	-	-	-	-	-	-	-	50	-
*Corytoplectus speciosus* **(H)**	-	-	-	-	-	-	-	-	-	-	-	-	-	-	-	-	-	-	-	-	-	-	50	-
*Clitoria pozuzoensis* **(C)**	-	-	-	-	-	-	-	-	-	-	-	-	-	-	-	-	-	-	-	-	-	-	50	-
*Sloanea ptariana* **(T)**	-	-	-	-	-	-	-	-	-	-	-	-	-	-	-	-	-	-	-	-	-	-	50	-
*Thelypteris patens* **(H)**	-	-	-	-	-	-	-	-	-	-	-	-	-	-	-	-	-	-	-	-	-	-	50	-
*Perebea angustifolia* **(T)**	-	-	-	-	-	-	-	-	-	-	-	-	-	-	-	-	-	-	-	-	-	-	50	-
*Miconia amplexicaulis* **(S)**	-	-	-	-	-	-	-	-	-	-	-	-	-	-	-	-	-	-	-	-	-	-	50	-
*Bauhinia tarapotensis* **(S)**	-	-	-	-	-	-	-	-	-	-	-	-	-	-	-	-	-	-	-	-	-	-	50	-
*Polybotrya caudata* **(H)**	-	-	-	-	-	-	-	-	-	-	-	-	-	-	-	-	-	-	-	-	-	-	50	-
*Piper arboreum* **(T)**	-	-	-	-	-	-	-	-	-	-	-	-	-	-	-	-	-	-	-	-	-	-	50	-
*Paullinia serjaniifolia* **(C)**	-	-	-	-	-	-	-	-	-	-	-	-	-	-	-	-	-	-	-	-	-	-	50	-
*Boehmeria pavonii* **(S)**	-	-	-	-	-	-	-	-	-	-	-	-	-	-	-	-	-	-	-	-	-	-	50	-
*Tectaria antioquoiana* **(H)**	-	-	-	-	-	-	-	-	-	-	-	-	-	-	-	-	-	-	-	-	-	-	50	-
***Pachiro brevipedis-Euterpetum catingae***																								
*Mauritiella aculeata* **(T)**	-	-	-	-	-	-	-	-	-	-	-	-	-	-	-	-	-	-	-	-	-	-	-	100
*Caraipa utilis* **(S)**	-	-	-	-	-	-	-	-	-	-	-	-	-	-	-	-	-	-	-	-	-	-	-	100
*Euterpe catinga* **(T)**	-	-	-	-	-	-	-	-	-	-	-	-	-	-	-	-	-	-	-	-	-	-	-	100
*Pachira brevipes* **(T)**	-	-	-	-	-	-	-	-	-	-	-	-	-	-	-	-	-	-	-	-	-	-	-	80
*Doliocarpus major* **(C)**	-	-	-	-	-	-	-	-	-	-	-	-	-	-	-	-	-	-	-	-	-	-	-	70
*Ocotea olivacea* **(T)**	-	-	-	-	-	-	-	-	-	-	-	-	-	-	-	-	-	-	-	-	-	-	-	60
*Clusia amazonica* **(T)**	-	-	-	-	-	-	-	-	-	-	-	-	-	-	-	-	-	-	-	-	-	-	25	60
*Amaioua corymbosa* **(S)**	-	-	-	-	-	-	-	-	-	-	-	-	-	-	-	-	-	-	-	-	-	-	-	60
*Cathedra acuminata* **(T)**	-	-	-	-	-	-	-	-	-	-	-	-	-	-	-	-	-	-	-	-	-	-	-	50
*Chrysophyllum* *bombycinum* **(T)**	-	-	-	-	-	-	-	-	-	-	-	-	-	-	-	-	-	-	-	-	-	-	-	50
*Mauritia carana* **(T)**	-	-	-	-	-	-	-	-	-	-	-	-	-	-	-	-	-	-	-	-	-	-	-	40
*Lindsaea divaricata* **(H)**	-	-	-	-	-	-	-	-	-	-	-	-	-	-	-	-	-	-	-	-	-	-	-	40
*Vantanea parviflora* **(T)**	-	-	-	-	-	-	-	-	-	-	-	-	-	-	-	-	-	-	-	-	-	-	-	30
**SUPRATROPICAL RAINFORESTS FROM SOUTHERN PERU WITH *POLYLEPIS (POLYLEPIDETEA TARAPACANO-BESSERI)***																								
*Bomarea ovata* **(C)**	100	-	-	-	33	-	-	-	-	-	-	-	-	-	-	-	-	-	-	-	-	-	-	-
*Berberis peruviana* **(S)**	100	-	-	-	33	-	-	-	-	-	-	-	-	-	-	-	-	-	-	-	-	-	-	-
*Peperomia hartwegiana* **(H)**	100	-	-	-	-	-	-	-	-	-	-	-	-	-	-	-	-	-	-	-	-	-	-	-
**MESO- SUPRA TROPICAL RAINFOREST WITH *OREOPANAX (PRUNO RIGIDAE-OREOPANACETEA FLORIBUNDI),* AND SHRUB FORMATIONS WITH *BACCHARIS (CLEMATIDO PERUVIANAE-BACCHARITETEA LATIFOLIAE)***																								
*Oreopanax eriocephalus* **(T)**	-	60	-	-	100	-	-	20	67	100	50	14	-	-	-	20	-	-	-	-	-	-	-	-
*Clematis haenkeana* **(C)**	-	40	-	-	14	-	-	-	-	80	25	43	50	-	-	20	-	-	-	-	-	-	-	-
*Hesperomeles ferruginea* **(T)**	-	-	-	-	100	-	-	-	-	60	50	-	-	-	13	-	-	-	-	-	-	-	-	-
*Myrcianthes myrsinoides* **(T)**	-	-	-	-	-	-	-	20	33	100	75	-	-	-	-	-	-	-	-	-	-	-	-	-
*Mauria heterophylla* **(S)**	-	-	-	-	-	-	-	-	33	80	50	-	-	-	-	-	-	-	-	-	-	-	-	-
*Adiantum concinnum* **(H)**	-	-	-	-	-	-	-	20	67	60	-	-	-	-	-	20	-	-	-	-	-	-	-	-
*Oreocallis grandiflora* **(S)**	-	-	-	-	-	-	-	40	-	60	25	-	-	-	-	-	-	-	-	-	-	-	-	-
*Vallea stipularis* **(S)**	-	-	-	-	-	-	-	20	33	-	-	29	-	-	-	-	-	-	-	-	20	-	-	-
*Baccharis latifolia* **(S)**	-	-	-	-	-	-	-	-	-	-	25	100	50	-	-	-	-	-	-	-	-	-	-	-
*Berberis beauverdiana* **(S)**	-	-	-	-	-	-	-	-	-	100	-	-	50	-	-	-	-	-	-	-	-	-	-	-
*Delostoma integrifolium* **(T)**	-	-	-	-	-	-	-	40	33	-	-	-	-	-	-	-	-	-	-	-	-	-	-	-
*Solanum oblongifolium* **(S)**	-	-	-	-	-	-	-	40	33	-	-	-	-	-	-	-	-	-	-	-	-	-	-	-
*Oreopanax aff cuspidatus* **(T)**	-	-	-	-	-	-	-	20	67	40	25	-	-	-	-	-	-	25	-	-	-	-	-	-
*Oxalis peduncularis* **(C)**	-	-	-	-	-	-	-	-	33	60	-	-	-	-	-	-	-	-	-	-	-	-	-	-
*Salvia punctate* **(SS)**	-	-	-	-	-	-	-	-	33	-	75	-	-	-	-	-	-	-	-	-	-	-	-	-
*Mutisia acuminata* **(C)**	-	-	-	-	-	-	-	-	33	-	25	-	-	-	-	-	-	-	-	-	-	-	-	-
*Lupinus mutabilis* **(S)**	-	-	-	-	-	-	-	-	67	-	50	-	50	-	-	-	-	-	-	-	-	-	-	-
*Citharexylum flexuosum* **(S)**	-	-	-	-	-	-	-	60	-	80	-	-	-	-	-	-	-	-	-	-	-	-	-	-
*Monactis flaverioides* **(S)**	-	-	-	-	-	-	-	-	33	40	-	100	100	-	13	-	-	-	-	-	-	-	-	-
*Prunus rigida* **(T)**	-	-	-	-	-	-	-	-	-	80	-	-	50	-	-	-	-	-	-	-	-	-	-	-
*Clusia pseudomangle* **(S)**	-	-	-	-	-	-	-	40	-	-	-	-	-	-	-	-	-	50	-	-	-	-	-	-
*Fuchsia ayavacensis* **(S)**	-	-	-	-	-	-	-	-	40	67	-	-	-	-	-	-	-	-	-	-	-	-	-	-
*Monnina pilosa* **(S)**	-	-	-	-	-	-	-	20	33	100	50	-	-	-	-	20	-	-	-	-	-	-	-	-
*Lepechinia mollis* **(S)**	-	-	-	-	-	-	-	-	-	40	50	-	-	-	-	-	-	-	-	-	-	-	-	-
*Otholobium munyense* **(S)**	-	20	-	-	-	-	-	-	-	-	25	43	100	-	-	-	-	-	-	-	-	-	-	-
*Hedyosmum scabrum* **(T)**	-	-	-	-	-	-	-	40	33	-	-	-	-	-	13	20	-	25	-	-	-	-	-	-
*Miconia denticulate* **(S)**	-	-	-	-	-	-	-	100	67	80	-	-	-	-	-	-	-	-	-	-	-	-	-	-
*Ilex uniflora* **(T)**	-	-	-	-	-	-	-	60	67	80	-	-	50	-	-	-	-	-	-	-	-	-	-	-
*Aphelandra acanthifolia* **(S)**	-	-	-	-	-	-	-	40	33	60	-	-	-	-	-	-	-	-	-	-	-	-	-	-
*Cronquistianthus marrubiifolius* **(S)**	-	-	-	-	-	-	-	40	33	80	75	-	-	-	-	-	-	-	-	-	-	-	-	-
*Passiflora sagasteguii* **(C)**	-	-	-	-	-	-	-	60	33	80	75	-	-	-	-	-	-	-	-	-	-	-	-	-
*Clusia multiflora* **(T)**	-	-	-	-	-	-	-	40	67	80	-	-	-	-	25	-	-	-	33	-	-	-	-	-
*Myrsine latifolia* **(T)**	-	-	-	-	-	-	-	40	33	100	-	-	-	-	-	20	100	-	-	-	-	-	-	-
*Croton abutiloides* **(S)**	-	-	-	-	-	-	-	20	-	60	-	-	-	-	-	60	-	-	-	-	-	-	-	-
*Frangula sphaerosperma* **(S)**	-	-	-	-	-	-	-	20	-	40	-	-	50	-	-	-	-	-	-	-	-	-	-	-
**THERMO-MESOTROPICAL FORESTS WITH *ALNUS* (*ALNETEA ACUMINATAE*)**																								
*Alnus acuminata* **(T)**	-	-	-	-	-	-	-	-	-	-	-	-	-	-	50	20	100	25	-	-	20	-	-	-
*Pteris deflexa* **(H)**	-	-	-	-	-	-	-	-	-	-	-	-	-	-	50	40	-	-	-	-	-	-	-	-
*Escallonia pendula* **(T)**	-	-	-	-	-	-	-	-	-	-	-	-	-	-	-	100	50	25	-	-	-	-	-	-
*Thelypteris pennata* **(H)**	-	-	-	-	-	17	-	-	-	-	-	-	-	-	-	60	-	50	-	-	-	-	-	-
*Pentacalia reflexa* **(S)**	-	-	-	-	-	-	-	-	-	-	-	-	-	-	-	-	100	75	-	-	-	-	-	-
*Bia alienata* **(C)**	-	-	-	-	-	-	-	-	-	-	-	-	-	-	-	-	100	50	-	-	-	-	-	-
**THERMOTROPICAL RAINFOREST FROM SOUTHERN PERU *(SERPOCAULO DASYPLEURONIS-ALCHORNEION LATIFOLIAE* all. nov.)**																								
*Lasiacis ligulata* **(H)**	-	-	71	-	-	-	-	-	-	-	-	-	-	-	-	-	-	-	-	-	-	100	25	-
*Alchornea latifolia* **(S)**	-	-	57	43	-	-	-	-	-	-	-	-	-	-	-	-	-	-	-	-	-	-	-	-
*Psychotria poeppigiana* **(S)**	-	-	43	-	-	-	-	-	-	-	-	-	-	-	-	-	-	-	-	-	-	-	-	-
*Serpocaulon dasypleuron* **(H)**	-	-	80	71	-	-	-	-	-	-	-	-	-	-	-	-	-	-	-	-	-	-	-	-
*Lindsaea arcuata* **(H)**	-	40	-	29	-	67	-	-	-	-	-	-	-	-	-	-	-	-	-	-	-	-	-	-
*Cyathea delgadii* **(T)**	-	-	86	-	-	-	-	-	-	-	-	-	-	-	13	-	-	-	67	-	-	-	-	-
*Olyra latifolia* **(H)**	-	-	86	-	-	50	-	-	-	-	-	-	-	-	-	-	-	-	-	-	-	-	-	-
*Graffenrieda cucullata* **(T)**	-	-	86	-	-	-	-	-	-	-	-	-	-	-	25	-	-	-	67	100	40	-	-	-
*Trema micrantha* **(T)**	-	-	-	57	-	-	-	-	-	-	-	-	-	-	-	-	-	-	-	-	-	-	-	-
**THERMO- INFRATROPICAL RAINFORESTS FROM NORTHERN PERU *(PTERIDI CRETICAE-CYATHEION CARACASANAE* all. nov.)**																								
*Pteris cretica* **(H)**	-	-	-	14	-	67	100	-	-	-	-	-	-	-	-	-	-	-	-	-	-	-	-	-
*Pennisetum peruvianum* **(H)**	-	-	-	-	-	67	-	-	-	-	-	-	-	-	-	-	-	-	-	-	-	100	-	-
*Cyrtocymura scorpioides* **(S)**	-	-	-	-	-	33	-	-	-	-	-	-	-	-	-	-	-	-	33	-	-	-	-	-
*Fuchsia mathewsii* **(S)**	-	-	-	-	-	33	100	-	-	-	-	-	-	-	-	-	-	-	-	-	-	-	-	-
*Cecropia utcubambana* **(T)**	-	-	-	-	-	33	-	-	-	-	-	-	-	-	-	-	-	-	-	17	-	100	50	-
*Seemannia sylvatica* **(H)**	-	-	-	-	-	33	-	-	-	-	-	-	-	-	-	20	-	-	-	33	-	-	-	-
*Miconia adinantha* **(S)**	-	-	-	-	-	33	-	40	-	-	-	-	-	-	-	-	-	50	-	-	-	-	-	-
*Begonia peruviana* **(C)**	-	-	-	-	-	33	-	-	-	-	-	-	-	-	13	-	-	-	67	67	-	-	-	-
*Piper lineatum* **(S)**	-	-	14	-	-	33	-	-	-	-	-	-	-	-	-	100	-	25	-	-	20	-	-	-
**MESO- TO INFRATROPICAL RAINFORESTS FROM CENTRAL PERU *(SANCHEZIO OBLONGAE-HEDYOSMION RACEMOSAE* all. nov.)**																								
*Piper chanchamayanum* **(S)**	-	-	-	-	-	-	-	-	-	-	-	-	-	-	-	-	-	-	100	33	-	-	-	-
*Zeugites americanus* **(H)**	-	-	-	-	-	-	-	-	-	-	-	-	-	-	-	-	-	-	67	17	-	100	-	-
*Miconia affinis* **(T)**	-	-	-	-	-	-	-	-	-	-	-	-	-	-	-	-	-	-	33	83	-	-	-	-
*Sanchezia oblonga* **(S)**	-	-	-	-	-	-	-	-	-	-	-	-	-	-	-	-	-	-	33	83	-	25	-	-
*Geonoma stricta* **(S)**	-	-	-	-	-	-	-	-	-	-	-	-	-	-	-	-	-	-	33	50	-	-	-	-
*Dennstaedtia auriculata* **(H)**	-	-	-	-	-	-	-	-	-	-	-	-	-	-	-	-	-	-	33	50	-	-	-	-
*Gurania lobate* **(C)**	-	-	-	-	-	-	-	-	-	-	-	-	-	-	-	-	-	-	33	50	-	-	-	-
*Anthurium aff amoenum* **(H)**	-	-	-	-	-	-	-	-	-	-	-	-	-	-	-	-	-	-	33	50	-	-	-	-
*Liabum nudicaule* **(S)**	-	-	-	-	-	-	-	-	-	-	-	-	-	-	-	-	-	-	33	50	-	-	25	-
*Blepharodon salicinus* **(C)**	-	-	-	-	-	-	-	-	-	-	-	-	-	-	-	-	-	-	33	33	-	-	-	-
*Weinmannia glomerata* **(T)**	-	-	-	-	-	-	-	-	-	-	-	-	-	-	-	-	-	-	33	-	80	-	-	-
*Baccharis decussata subsp. jelskii* **(S)**	-	-	-	-	-	-	-	-	-	-	-	-	-	-	-	-	-	-	33	-	60	-	-	-
*Pilea haenkei* **(S)**	-	-	-	-	-	-	-	-	-	-	-	-	-	-	-	-	-	-	33	17	-	100	-	-
*Piper malifolium* **(T)**	-	-	-	-	-	-	-	-	-	-	-	-	-	-	-	-	-	-	33	17	-	100	-	-
*Passiflora rubra* **(C)**	-	-	-	-	-	-	-	-	-	-	-	-	-	-	-	-	-	-	33	-	20	-	50	-
*Pteris podophylla* **(H)**	-	-	-	-	-	-	-	-	-	-	-	-	-	-	-	-	-	-	100	83	-	-	25	-
*Fuchsia ovalis* **(S)**	-	-	-	-	-	-	-	-	-	-	-	-	-	-	-	-	-	-	67	33	-	-	-	-
*Hedyosmum racemosum* **(T)**	-	-	-	-	-	-	-	-	-	-	-	-	-	-	-	-	-	-	67	83	-	-	-	-
*Oreopanax polycephalus* **(T)**	-	-	-	-	-	-	-	-	-	-	-	-	-	-	-	-	-	-	67	50	-	-	-	-
*Pteris quadriaurita* **(H)**	-	-	-	-	-	-	-	-	-	-	-	-	-	-	25	20	-	-	33	-	40	-	-	-
*Anthurium breviscapum* **(H)**	-	-	-	-	-	-	-	-	-	-	-	-	-	-	-	-	-	-	67	100	20	-	-	-
*Monstera obliqua* **(H)**	-	-	-	-	-	-	-	-	-	-	-	-	-	-	-	-	-	-	33	17	-	100	25	-
*Clusia trochiformis* **(S)**	-	-	-	-	-	-	-	-	-	-	-	-	-	-	-	-	-	-	-	10	40	100	-	-
*Miconia cyanocarpa* **(S)**	-	-	-	-	-	-	-	-	-	-	-	-	-	-	-	-	-	-	-	-	80	100	-	-
*Monnina marginata* **(S)**	-	-	-	-	-	-	-	-	-	-	-	-	-	-	-	-	-	-	-	-	40	-	50	-
*Niphidium albopunctatissimum* **(H)**	-	-	-	-	-	-	-	-	-	-	-	-	-	-	-	-	-	-	-	-	40	100	-	-
*Dryopteris wallichiana* **(H)**	-	-	-	-	-	-	-	-	-	-	-	-	-	-	-	-	-	25	-	-	20	100	-	-
*Cedrela fissilis* **(T)**	-	-	-	-	-	-	-	-	-	-	-	-	-	-	-	-	-	-	-	67	-	-	25	-
*Palicourea guianensis* **(S)**	-	-	-	-	-	-	-	-	-	-	-	-	-	-	-	-	-	-	-	33	-	-	50	-
*Iresine diffusa* **(C)**	-	-	-	-	-	-	-	-	-	-	-	-	-	-	-	-	-	-	33	33	20	-	50	-
*Diplazium tungurahuae* **(H)**	-	-	-	-	-	-	-	-	-	-	-	-	-	-	-	-	-	-	33	33	-	-	25	-
*Inga semialata* **(T)**	-	-	-	14	-	-	-	-	-	-	-	-	-	-	-	-	-	-	-	-	-	-	25	60
*Piper aduncum* **(S)**	-	-	-	14	-	-	-	-	-	-	-	-	-	-	-	-	-	-	-	-	-	-	50	-
*Inga edulis* **(T)**	-	-	-	-	-	-	-	-	-	-	-	-	-	-	-	-	-	-	-	-	-	-	75	-
**MESO- TO INFRATROPICAL MOUNTAIN RAINFORESTS FROM PERU *(MORELLO PUBESCENTIS-MYRSINETEA CORIACEAE* cl. nov.)**																								
*Niphidium crassifolium* **(H)**	-	60	29	14	-	-	-	20	-	20	-	-	50	-	50	20	100	50	67	33	40	-	-	-
*Blechnum occidentale* **(H)**	-	-	-	-	-	-	-	100	67	60	-	-	-	-	-	40	50	75	100	50	80	100	50	-
*Pteridium arachnoideum* **(H)**	-	-	-	14	33	83	-	60	-	80	-	-	-	-	13	40	-	25	-	-	20	-	-	-
*Baccharis inamoena* **(S)**	-	80	-	-	100	67	100	-	-	-	-	-	-	-	13	20	100	100	-	-		-	-	-
*Ageratina sternbergiana* **(S)**	-	40	-	-	-	-	-	40	33	60	100	43	-	-	25	-	50	-	-	-		-	-	-
*Myriocarpa stipitata* **(T)**	-	20	-	29	-	17	100	-	-	-	-	-	-	-	-	80	-	-	67	-	-	-	50	-
*Tradescantia cymbispatha* **(H)**	-	40	-	43	-	17	-	20	-	-	-	-	-	-	13	40	-	50	-	-	-	-	-	-
*Condaminea corymbosa* **(S)**	-	-	-	14	-	50	-	-	-	-	-	-	-	-	50	-	-	-	33	20	100	75	-	-
*Ctenitis sloanei* **(H)**	-	-	-	14	-	-	-	-	-	-	-	-	-	-	63	20	-	-	100	17	80	-	50	-
*Solanum maturecalvans* **(S)**	-	60	-	14	67	-	-	40	33	-	-	-	-	-	-	-	-	-	33	-	-	-	-	-
*Saurauia peruviana* **(T)**	-	-	-	29	-	-	-	-	33	-	-	-	-	-	-	-	-	-	33	50	20	100	-	-
*Cavendishia bracteata* **(S)**	-	-	-	14	-	-	-	-	-	-	-	-	-	-	25	-	-	-	33	17	20	-	-	-
*Serpocaulon caceresii* **(H)**	-	80	57	14	-	17	-	-	-	-	-	-	-	-	-	-	-	-	-	17	-	-	-	-
*Siparuna aspera* **(S)**	-	20	29	-	-	83	-	-	-	-	-	-	-	-	-	-	-	75	-	-	-	-	25	-
*Conostegia inusitata* **(T)**	-	20	-	43	-	-	-	-	-	-	-	-	-	-	38	40	-	75	-	-	-	-	-	-
*Alchornea glandulosa* **(T)**	-	-	43	-	-	17	-	-	-	-	-	-	-	-	-	50	20	-	-	-	17	-	-	-
*Palicourea amethystine* **(S)**	-	-	-	-	-	-	-	60	-	-	-	-	-	-	13	-	-	-	67	50	40	-	-	-
*Myrsine coriacea* **(T)**	-	-	-	-	-	-	-	-	-	60	-	14	-	-	-	-	-	75	33	83	-	-	-	-
*Morella pubescens* **(T)**	-	-	-	14	-	-	-	-	-	-	-	-	-	-	50	-	-	-	33	33	60	-	-	-
*Cyathea caracasana* **(T)**	-	-	-	-	-	33	-	-	-	-	-	-	-	-	-	-	-	-	33	83	20	-	25	-
*Munnozia hastifolia* **(S)**	-	-	-	-	-	-	-	-	-	-	-	-	-	-	50	20	-	-	33	17	-	-	25	-
*Nephrolepis cordifolia* **(H)**	-	-	29	-	-	33	-	-	-	-	-	-	-	-	-	60	-	-	-	-	-	-	50	-
*Heliocarpus americanus* **(T)**	-	-	-	-	-	67	-	-	-	-	-	-	-	-	13	-	-	-	-	100	-	100	25	-
*Cinchona pubescens* **(T)**	-	-	-	-	-	67	-	-	-	-	-	-	-	-	50	-	-	-	-	17	-	100	-	-
*Ipomoea sp.* **(C)**	-	20	-	-	-	-	100	-	-	-	-	-	-	-	-	-	-	25	67	-	-	-	-	-
*Vismia tomentosa* **(T)**	-	-	71	14	-	-	-	-	-	-	-	-	-	-	-	-	-	-	-	67	-	-	-	-
*Begonia cyathophora* **(H)**	-	-	-	-	100	-	-	-	-	-	-	-	-	-	-	-	-	-	-	-	-	100	25	-
*Smilax domingensis* **(C)**	-	-	-	-	-	50	-	-	-	-	-	-	-	-	-	-	-	50	-	-	-	100	-	-
*Blechnum cordatum* **(H)**	-	-	-	-	-	-	-	-	-	-	-	-	-	-	13	-	-	-	33	-	40	-	-	-
*Disterigma alaternoides* **(SS)**	-	-	-	-	-	-	-	-	-	-	-	-	-	-	13	-	-	-	33	-	20	-	-	-
*Schefflera acuminata* **(T)**	-	-	-	-	-	-	-	-	-	-	-	-	-	-	38	-	-	-	33	50	20	-	-	-
*Ochroma pyramidale* **(T)**	-	-	-	-	-	-	-	-	-	-	-	-	-	-	-	40	-	-	-	-	-	100	100	-
*Festuca sp.* **(H)**	-	-	-	-	67	-	-	-	-	-	50	-	-	-	-	-	-	-	-	-	-	-	-	-
*Dioscorea altissima* **(C)**	-	-	-	-	-	-	-	-	-	-	-	-	-	-	-	80	-	-	-	-	-	-	-	60
*Polystichum montevidense* **(H)**	-	40	-	-	-	-	-	-	-	-	-	-	-	-	-	-	-	-	-	-	40	-	-	-

Values in cells are percentage frequencies. Underlined numbers indicate species with a frequency percentage >30% and a phi measure >0.2. Only the most frequent and characteristic species are shown. The letters in brackets near the species are their life forms: T = Tree, S = Shrub, SS = Subshrub, C = Climber, Ep = Epiphyte, H = Herb. The colored squares are diagnostic species: Brown = *Polylepidetea tarapacano-besseri,* red = *Pruno rigidae-Oreopanacetea floribundi,* grey = *Clematido peruvianae-Baccharitetea latifoliae,* blue = *Alnetalia acuminatae, Alnetea acuminatae,* orange = *Serpocaulo dasypleuronis-Alchorneion latifoliae,* pink = *Pteridi creticae-Cyatheion caracasanae,* green: *Sanchezio oblongae-Hedyosmion racemosi.* Localities. 1—Ollachea, Puno (author data). 2—Below Ollachea, Camatani, Puno (author data). 3—San Isidro bridge, San Gabán, Puno (author data). 4—Payachaca bridge, below Ollachea, Puno (author data). 5—Ollachea, Puno (author data). 6—La Coipa, Cajamarca (author data). 7—Tamborapa, Cajamarca (author data). 8—Cachil, Cajamarca [18]. 9—Cachil, road down to Guzmango, Cajamarca [18]. 10—Cachil and San Miguel, Cajamarca; Cascas, La Libertad [18]. 11—Contumazá, road down to Guzmango, Cajamarca [18]. 12—Morocha, Bellavista and Hualgayoc, Cajamarca [18]. 13—Cachil, Cajamarca [18]. 14—Jenaro Herrera, Loreto [64]. 15—San Pedro de la Capilla, Cajamarca (author data). 16—Gocta, Chachapoyas, Amazonas (author data). 17—Ocol, Chachapoyas, Amazonas (author data). 18—Ocol, Chachapoyas, Amazonas (author data). 19—Satipo, Junín (author data). 20—Satipo, Junín (author data). 21—Below Carpish, Huánuco (author data). 22—Below Carpish, Huánuco (author data). 23—Tingo María, Huánuco (author data). 24—Alpahuayo-Mishana, Iquitos, Loreto [65].

**Table A2:** Holotypes of the new associations described.

Association Number in Text	2	3	4	5	6	8	9	10	11	12	13	14	16
Vegetation canopy (m)	10	25	20	8	20	28	20	15	34	20	25	10	26
Slope (%)	60	0	70	0	40	20	20	20	0	30	10	50	40
Altitude (m)	2509	791	1230	2774	1689	2664	1783	2337	2378	2358	1351	2533	894
Orientation	SE	-	E	-	NW	S	SW	S	S	S	E	W	NE
Area (m^2^)	200	200	100	100	200	500	500	100	500	200	1000	400	500
***Smallantho parvicipitis-Oreopanacetum eriocephali* ass. nov.**													
*Ageratina sternbergiana* **(S)**	+	-	-	-	-	-	-	1	-	-	-	-	-
*Oreopanax eriocephalus* **(T)**	1	-	-	1	-	-	-	-	-	-	-	-	-
*Tradescantia cymbispatha* **(H)**	2	-	-	-	-	-	1	-	2	-	-	-	-
*Serpocaulon caceresii* **(H)**	2	1	-	-	-	-	-	-	-	-	-	-	-
*Solanum maturecalvans* **(S)**	1	-	-	-	-	-	-	-	-	-	-	-	-
*Miconia aff adinantha* **(S)**	1	-	-	-	-	-	-	-	-	-	-	-	-
*Serpocaulon dasypleuron* **(H)**	1	1	-	-	-	-	-	-	-	-	-	-	-
*Begonia bracteosa* **(H)**	1	-	-	-	-	-	-	-	-	-	-	-	-
*Smallanthus parviceps* **(T)**	4	-	-	-	-	-	-	-	-	-	-	-	-
*Liabum solidagineum* **(S)**	2	-	-	-	-	-	-	-	-	-	-	-	-
*Fuchsia boliviana* **(S)**	1	-	-	-	-	-	-	-	-	-	-	-	-
*Melica scabra* **(H)**	1	-	-	-	-	-	-	-	-	-	-	-	-
*Rubus boliviensis* **(S)**	+	-	-	-	-	-	-	-	-	-	-	-	-
*Solanum aphyodendron* **(T)**	3	-	-	-	-	-	-	-	-	-	-	-	-
*Phenax angustifolius* **(T)**	1	-	-	-	-	-	-	-	-	-	-	-	-
*Croton regelianus* **(S)**	+	-	-	-	-	-	-	-	-	-	-	-	-
*Pityrogramma calomelanos* **(H)**	+	-	-	-	-	-	-	-	-	-	-	-	-
*Polystichum montevidense* **(H)**	2	-	-	-	-	-	-	-	-	-	-	1	-
*Pennisetum latifolium* **(H)**	1	-	-	-	-	-	-	-	-	-	-	-	-
*Lozanella enantiophylla* **(T)**	3	-	-	-	-	-	-	-	-	-	-	-	-
*Equisetum sp._sg1* **(H)**	1	-	-	-	-	-	-	-	-	-	-	-	-
*Melastomataceae 01_sg1* **(S)**	3	-	-	-	-	-	-	-	-	-	-	-	-
*Galium hypocarpium* **(H)**	1	-	-	-	-	-	-	-	-	-	-	-	-
***Iriartello setigerae-Cinchonetum micranthae* ass. nov.**													
*Siparuna aspera* **(S)**	-	1	-	-	1	-	-	-	3	-	-	-	1
*Olyra latifolia* **(H)**	-	2	-	-	1	-	-	-	-	-	-	-	-
*Alchornea glandulosa* **(T)**	-	2	-	-	-	-	1	-	-	-	-	-	-
*Graffenrieda cucullata* **(T)**	-	3	-	-	-	-	-	-	-	4	3	2	-
*Cyathea delgadii* **(T)**	-	3	-	-	-	-	-	-	-	2	-	-	-
*Alchornea latifolia* **(S)**	-	2	-	-	-	-	-	-	-	-	-	-	-
*Cinchona micrantha* **(T)**	-	4	-	-	-	-	-	-	-	-	-	-	-
*Iriartella setigera* **(S)**	-	2	-	-	-	-	-	-	-	-	-	-	-
*Heliconia hirsute* **(H)**	-	+	-	-	-	-	-	-	-	-	-	-	-
*Vismia tomentosa* **(T)**	-	1	-	-	-	-	-	-	-	-	-	-	-
*Cyathea subincisa* **(T)**	-	1	-	-	-	-	-	-	-	-	-	-	-
*Philodendron rudgeanum* **(Ep)**	-	1	-	-	-	-	-	-	-	-	-	-	-
*Dieffenbachia humilis* **(H)**	-	+	-	-	-	-	-	-	-	-	-	-	-
*Miconia lourteigiana* **(T)**	-	1	-	-	-	-	-	-	-	-	-	-	-
*Aphelandra aurantiaca* **(S)**	-	1	-	-	-	-	-	-	-	-	-	-	-
*Blakea sawadae* **(C)**	-	1	-	-	-	-	-	-	-	-	-	-	-
*Palicourea fastigiata* **(S)**	-	1	-	-	-	-	-	-	-	-	-	-	-
*Liabum acuminatum* **(S)**	-	1	-	-	-	-	-	-	-	-	-	-	-
***Acalypho macrostachyae-Cecropietum polystachyae* ass. nov.**													
*Saurauia peruviana* **(T)**	-	-	1	-	-	-	-	-	-	-	2	1	-
*Cecropia polystachya* **(T)**	-	-	4	-	-	-	-	-	-	-	-	-	+
*Chusquea scandens* **(S)**	-	-	1	-	-	4	1	-	1	-	-	4	-
*Pteris cretica* **(H)**	-	-	1	-	-	-	-	-	-	-	-	-	-
*Morella pubescens* **(T)**	-	-	2	-	-	-	-	-	-	-	+	-	-
*Trema micrantha* **(T)**	-	-	1	-	-	-	-	-	-	-	-	-	-
*Anthurium grande* **(H)**	-	-	1	-	-	-	-	-	-	-	-	-	-
*Acalypha macrostachya* **(T)**	-	-	4	-	-	-	-	-	-	-	-	-	-
*Urera caracasana* **(T)**	-	-	2	-	-	-	-	-	-	-	-	-	-
*Lycianthes inaequilatera* **(S)**	-	-	1	-	-	-	-	-	-	-	-	-	-
*Bignoniaceae* **(C)**	-	-	+	-	-	-	+	-	-	-	1	-	-
*Psychotria trichostoma* **(S)**	-	-	3	-	-	-	-	-	-	-	-	-	-
*Philodendron divaricatum* **(C)**	-	-	1	-	-	-	-	-	-	-	-	-	-
*Peperomia pseudorhynchophoros* **(H)**	-	-	2	-	-	-	-	-	-	-	-	-	-
*Thelypteris brausei* **(H)**	-	-	+	-	-	-	-	-	-	-	-	-	-
***Crotono churumayensis-Hesperomeletum ferrugineae* ass. nov.**													
*Rubus praecox* **(S)**	-	-	-	1	-	-	-	-	1	-	-	-	-
*Baccharis inamoena* **(S)**	-	-	-	4	2	+	-	3	2	-	-	-	-
*Hesperomeles ferruginea* **(T)**	-	-	-	2	-	1	-	-	-	-	-	-	-
*Festuca sp.* **(H)**	-	-	-	1	-	-	-	-	-	-	-	-	-
*Cuphea cordata* **(S)**	-	-	-	3	-	-	-	-	-	-	-	-	-
*Croton churumayensis* **(T)**	-	-	-	3	-	-	-	-	-	-	-	-	-
*Begonia cyathophora* **(H)**	-	-	-	1	-	-	-	-	-	-	-	-	-
*Senna birostris* **(S)**	-	-	-	1	-	-	-	-	-	-	-	-	-
*Ditassa sp._sg* **(Ep)**	-	-	-	1	-	-	-	-	-	-	-	-	-
*Peperomia sp._sg158* **(H)**	-	-	-	1	-	-	-	-	-	-	-	-	-
***Viburno reticulati-Weinmannietum spruceanae* ass. nov.**													
*Pteridium arachnoideum* **(H)**	-	-	-	-	2	-	-	-	-	-	-	-	-
*Lindsaea arcuata* **(H)**	-	-	-	-	2	-	-	-	-	-	-	-	-
*Condaminea corymbosa* **(S)**	-	-	-	-	1	-	-	-	-	-	-	-	3
*Nephrolepis cordifolia* **(H)**	-	-	-	-	1	-	1	-	-	-	-	-	2
*Viburnum reticulatum* **(T)**	-	-	-	-	1	-	-	-	-	-	-	-	-
*Weinmannia spruceana* **(T)**	-	-	-	-	4	-	-	-	-	-	-	-	-
*Fuchsia mathewsii* **(S)**	-	-	-	-	1	-	-	-	-	-	-	-	-
*Seemannia sylvatica* **(H)**	-	-	-	-	+	-	-	-	-	-	1	-	-
*Pouzolzia poeppigiana* **(T)**	-	-	-	-	+	-	-	-	-	-	-	-	-
*Clusia crenata* **(T)**	-	-	-	-	1	-	-	-	-	-	-	-	-
*Pitcairnia paniculate* **(H)**	-	-	-	-	+	-	-	-	-	-	-	-	-
*Anthurium hamiltonii* **(H)**	-	-	-	-	1	-	-	-	-	-	-	-	-
*Elaphoglossum aff andicola* **(H)**	-	-	-	-	1	-	-	-	-	-	-	-	-
*Myrsine manglilla* **(S)**	-	-	-	-	1	-	-	-	-	-	-	-	-
***Hypolepido parallelogrammae-Alnetum acuminatae* ass. nov.**													
*Niphidium crassifolium* **(H)**	-	-	-	-	-	1	-	+	+	+	+	1	-
*Munnozia hastifolia* **(S)**	-	-	-	-	-	+	-	-	-	-	-	-	-
*Alnus acuminata* **(T)**	-	-	-	-	-	3	-	4	+	-	-	1	-
*Ctenitis sloanei* **(H)**	-	-	-	-	-	1	-	-	-	3	-	2	2
*Hypolepis parallelogramma* **(H)**	-	-	-	-	-	1	-	-	-	-	-	-	-
*Solanum sp. 03* **(S)**	-	-	-	-	-	+	-	-	-	-	-	-	-
*Hypolepis obtusata* **(H)**	-	-	-	-	-	2	-	-	-	-	-	-	-
*Cyathea lechleri* **(T)**	-	-	-	-	-	1	-	-	-	-	-	-	-
*Aulonemia longiaristata* **(H)**	-	-	-	-	-	2	-	-	-	-	-	-	-
*Viburnum incarum* **(S)**	-	-	-	-	-	+	-	-	-	-	-	-	-
*Cronquistianthus sp. 01_cut* **(S)**	-	-	-	-	-	+	-	-	-	-	-	-	-
*Escallonia myrtilloides* **(S)**	-	-	-	-	-	1	-	-	-	-	-	-	-
*Weinmannia cymbifolia* **(S)**	-	-	-	-	-	1	-	-	-	-	-	-	-
*Ageratina neblinense* **(S)**	-	-	-	-	-	+	-	-	-	-	-	-	-
*Clusia ducu* **(T)**	-	-	-	-	-	2	-	-	-	-	-	-	-
*Psidium sp._cut7* **(T)**	-	-	-	-	-	1	-	-	-	-	-	-	-
*Rubiaceae sp. 01_cut7* **(S)**	-	-	-	-	-	+	-	-	-	-	-	-	-
*Tree shiny lanceolate leaves_cut7* **(T)**	-	-	-	-	-	+	-	-	-	-	-	-	-
*Desfontainia spinosa* **(S)**	-	-	-	-	-	1	-	-	-	-	-	-	-
*Hedyosmum translucidum* **(T)**	-	-	-	-	-	3	-	-	-	-	-	-	-
*Pleurothallis cordata* **(Ep)**	-	-	-	-	-	3	-	-	-	-	-	-	-
***Lueheo divaricatae-Ceroxylonetum peruviani* ass. nov.**													
*Dioscorea altissima* **(C)**	-	-	-	-	-	-	1	-	-	-	-	-	-
*Piper lineatum* **(S)**	-	-	-	-	-	-	1	-	-	-	-	-	-
*Adiantum concinnum* **(H)**	-	-	-	-	-	-	1	-	-	-	-	-	-
*Escallonia pendula* **(T)**	-	-	-	-	-	-	4	1	-	-	-	-	-
*Pteris deflexa* **(H)**	-	-	-	-	-	-	1	-	-	-	-	-	-
*Luehea divaricata* **(T)**	-	-	-	-	-	-	3	-	-	-	-	-	-
*Serpocaulon fraxinifolium* **(H)**	-	-	-	-	-	-	+	-	-	-	-	-	-
*Styloceras laurifolium* **(S)**	-	-	-	-	-	-	3	-	-	-	-	-	-
*Pteris quadriaurita* **(H)**	-	-	-	-	-	-	1	-	-	1	-	2	-
*Asplenium cuneatum* **(H)**	-	-	-	-	-	-	+	-	-	-	-	-	-
*Asplenium alatum* **(H)**	-	-	-	-	-	-	1	-	-	-	-	-	-
*Lycianthes sp._got5* **(S)**	-	-	-	-	-	-	1	-	-	-	-	-	-
*Miconia sp. 01_got5* **(S)**	-	-	-	-	-	-	1	-	-	-	-	-	-
*Sclerophyllous tree* *broad leaves_got5* **(T)**	-	-	-	-	-	-	2	-	-	-	-	-	-
*Ceroxylon peruvianum* **(T)**	-	-	-	-	-	-	3	-	-	-	-	-	-
***Mutisio wurdackii-Alnetum acuminatae* ass. nov.**													
*Blechnum occidentale* **(H)**	-	-	-	-	-	-	-	2	2	1	-	1	-
*Myrsine latifolia* **(T)**	-	-	-	-	-	-	-	2	-	-	-	-	-
*Pentacalia reflexa* **(S)**	-	-	-	-	-	-	-	+	-	-	-	-	-
*Bia alienate* **(C)**	-	-	-	-	-	-	-	1	-	-	-	-	-
*Mutisia wurdackii* **(C)**	-	-	-	-	-	-	-	2	1	-	-	-	-
*Weinmannia costulata* **(S)**	-	-	-	-	-	-	-	1	-	-	-	-	-
*Cortaderia jubata* **(H)**	-	-	-	-	-	-	-	+	-	-	-	-	-
***Axinaeo tomentosae-Ceroxylonetum quindiuensis* ass. nov.**													
*Conostegia inusitata* **(T)**	-	-	-	-	-	-	-	-	1	-	-	-	-
*Myrsine coriacea* **(T)**	-	-	-	-	-	-	-	-	1	-	2	-	-
*Cedrela odorata* **(T)**	-	-	-	-	-	-	-	-	3	-	-	-	-
*Miconia adinantha* **(S)**	-	-	-	-	-	-	-	-	2	-	-	-	-
*Cronquistianthus chachapoyensis* **(S)**	-	-	-	-	-	-	-	-	+	-	-	-	-
*Ceroxylon quindiuense* **(T)**	-	-	-	-	-	-	-	-	1	-	-	-	-
*Solanum barbulatum* **(S)**	-	-	-	-	-	-	-	-	+	-	-	-	-
*Axinaea tomentosa* **(S)**	-	-	-	-	-	-	-	-	+	-	-	-	-
*Vismia pozuzoensis* **(T)**	-	-	-	-	-	-	-	-	1	-	-	-	-
*Hedyosmum lechleri* **(T)**	-	-	-	-	-	-	-	-	4	-	-	-	-
*Palicourea stipularis* **(S)**	-	-	-	-	-	-	-	-	1	-	-	-	-
*Begonia sp._ocol13* **(H)**	-	-	-	-	-	-	-	-	1	-	-	-	-
*Dryopteris wallichiana* **(H)**	-	-	-	-	-	-	-	-	1	-	-	-	-
*Hydrangea jelskii* **(S)**	-	-	-	-	-	-	-	-	2	-	-	-	-
***Piperi chanchamayani-Cecropietum strigosae* ass. nov.**													
*Cecropia angustifolia* **(T)**	-	-	-	-	-	-	-	-	-	1	-	-	-
*Cyathea caracasana* **(T)**	-	-	-	-	-	-	-	-	-	1	1	-	1
*Anthurium breviscapum* **(H)**	-	-	-	-	-	-	-	-	-	1	1	-	-
*Diplazium tungurahuae* **(H)**	-	-	-	-	-	-	-	-	-	1	-	-	1
*Iresine diffusa* **(C)**	-	-	-	-	-	-	-	-	-	1	-	1	+
*Cecropia strigose* **(T)**	-	-	-	-	-	-	-	-	-	3	-	-	-
*Pteris podophylla* **(H)**	-	-	-	-	-	-	-	-	-	1	2	-	-
*Centropogon altus* **(S)**	-	-	-	-	-	-	-	-	-	2	-	-	-
*Pennisetum aff peruvianum* **(H)**	-	-	-	-	-	-	-	-	-	2	-	-	-
*Piper chanchamayanum* **(S)**	-	-	-	-	-	-	-	-	-	1	-	-	-
*Clusia aff pallida* **(T)**	-	-	-	-	-	-	-	-	-	1	-	-	-
*Ageratina tambillensis* **(S)**	-	-	-	-	-	-	-	-	-	+	-	-	-
*Dennstaedtia auriculata* **(H)**	-	-	-	-	-	-	-	-	-	1	1	-	-
*Liabum nudicaule* **(S)**	-	-	-	-	-	-	-	-	-	1	-	-	+
*Miconia sp. 08_sat* **(S)**	-	-	-	-	-	-	-	-	-	+	-	-	-
*Pilea haenkei* **(S)**	-	-	-	-	-	-	-	-	-	1	+	-	-
*Piper malifolium* **(T)**	-	-	-	-	-	-	-	-	-	1	-	-	-
*Adianthum raddianum* **(H)**	-	-	-	-	-	-	-	-	-	+	-	-	-
*Geonoma stricta* **(S)**	-	-	-	-	-	-	-	-	-	1	+	-	-
*Gurania lobata* **(C)**	-	-	-	-	-	-	-	-	-	1	-	-	-
*Palicourea amethystina* **(S)**	-	-	-	-	-	-	-	-	-	+	-	-	-
***Cedrelo fissilis-Ficetum maximae* ass. nov.**													
*Heliocarpus americanus* **(T)**	-	-	-	-	-	-	-	-	-	-	1	-	-
*Begonia peruviana* **(C)**	-	-	-	-	-	-	-	-	-	-	1	-	-
*Cavendishia bracteata* **(S)**	-	-	-	-	-	-	-	-	-	-	1	-	-
*Serpocaulon loriceum* **(H)**	-	-	-	-	-	-	-	-	-	-	1	-	-
*Schefflera acuminata* **(T)**	-	-	-	-	-	-	-	-	-	-	+	-	-
*Begonia parviflora* **(C)**	-	-	-	-	-	-	-	-	-	-	+	1	-
*Heliconia subulate* **(H)**	-	-	-	-	-	-	-	-	-	-	1	-	-
*Vernonanthura patens* **(S)**	-	-	-	-	-	-	-	-	-	-	2	-	-
*Lastreopsis effuse* **(H)**	-	-	-	-	-	-	-	-	-	-	1	-	-
*Hedyosmum racemosum* **(T)**	-	-	-	-	-	-	-	-	-	-	4	-	-
*Cedrela fissilis* **(T)**	-	-	-	-	-	-	-	-	-	-	1	-	3
*Blepharodon salicinus* **(C)**	-	-	-	-	-	-	-	-	-	-	1	-	-
*Oreopanax polycephalus* **(T)**	-	-	-	-	-	-	-	-	-	-	+	-	-
*Miconia affinis* **(T)**	-	-	-	-	-	-	-	-	-	-	1	-	-
*Anthurium aff amoenum* **(H)**	-	-	-	-	-	-	-	-	-	-	+	-	-
*Sanchezia oblonga* **(S)**	-	-	-	-	-	-	-	-	-	-	3	-	-
*Ficus maxima* **(T)**	-	-	-	-	-	-	-	-	-	-	4	-	-
*Dieffenbachia sp. 02_sat* **(H)**	-	-	-	-	-	-	-	-	-	-	2	-	-
*Alsophila mostellaria* **(T)**	-	-	-	-	-	-	-	-	-	-	1	-	-
*Ocotea obovata* **(T)**	-	-	-	-	-	-	-	-	-	-	1	-	-
*Dioscorea sp. 01_sat* **(C)**	-	-	-	-	-	-	-	-	-	-	+	-	-
*Cyclanthus bipartitus* **(H)**	-	-	-	-	-	-	-	-	-	-	1	-	1
*Croton lechheri* **(T)**	-	-	-	-	-	-	-	-	-	-	2	-	-
*Palicourea guianensis* **(S)**	-	-	-	-	-	-	-	-	-	-	1	-	1
*Columnea inaquilatera* **(H)**	-	-	-	-	-	-	-	-	-	-	+	-	-
*Elaphoglossum latifolium* **(H)**	-	-	-	-	-	-	-	-	-	-	+	-	-
*Megalastrum pulverulentum* **(H)**	-	-	-	-	-	-	-	-	-	-	+	-	-
*Ficus cuatrecasasiana* **(T)**	-	-	-	-	-	-	-	-	-	-	1	-	-
*Melastomataceae 02_sat* **(S)**	-	-	-	-	-	-	-	-	-	-	+	-	-
*Siparuna tomentosa* **(S)**	-	-	-	-	-	-	-	-	-	-	1	-	-
*Sorocea sp._sat8* **(S)**	-	-	-	-	-	-	-	-	-	-	1	-	-
*Clusia aff tarmensis* **(T)**	-	-	-	-	-	-	-	-	-	-	+	-	-
***Cecropio albicantis-Weinmannietum glomeratae* ass. nov.**													
*Prestoea carderi* **(S)**	-	-	-	-	-	-	-	-	-	-	1	2	-
*Blechnum cordatum* **(H)**	-	-	-	-	-	-	-	-	-	-	-	2	-
*Weinmannia latifolia* **(T)**	-	-	-	-	-	-	-	-	-	-	-	1	-
*Disterigma alaternoides* **(SS)**	-	-	-	-	-	-	-	-	-	-	-	1	-
*Piper augustum* **(S)**	-	-	-	-	-	-	-	-	-	-	-	1	-
*Disterigma acuminatum* **(SS)**	-	-	-	-	-	-	-	-	-	-	-	1	-
*Weinmannia glomerata* **(T)**	-	-	-	-	-	-	-	-	-	-	-	4	-
*Anthurium linganii* **(H)**	-	-	-	-	-	-	-	-	-	-	-	+	-
*Bromelia sp._tin_1* **(H)**	-	-	-	-	-	-	-	-	-	-	-	+	-
*Clidemia sp._tin_1* **(S)**	-	-	-	-	-	-	-	-	-	-	-	+	-
*Munnozia silphioides* **(H)**	-	-	-	-	-	-	-	-	-	-	-	1	-
*Miconia cyanocarpa* **(S)**	-	-	-	-	-	-	-	-	-	-	-	+	-
*Tibouchina saxosa* **(SS)**	-	-	-	-	-	-	-	-	-	-	-	+	-
*Centropogon reflexus* **(C)**	-	-	-	-	-	-	-	-	-	-	-	1	-
*Centropogon hirtus* **(S)**	-	-	-	-	-	-	-	-	-	-	-	1	-
*Fuchsia macrophylla* **(S)**	-	-	-	-	-	-	-	-	-	-	-	1	-
*Oyedaea sp._tin_1* **(S)**	-	-	-	-	-	-	-	-	-	-	-	+	-
*Cecropia albicans* **(T)**	-	-	-	-	-	-	-	-	-	-	-	1	-
*Bocconia integrifolia* **(T)**	-	-	-	-	-	-	-	-	-	-	-	+	-
*Oxalis dolichopoda* **(C)**	-	-	-	-	-	-	-	-	-	-	-	1	-
*Clusiaceae_tin_1* **(S)**	-	-	-	-	-	-	-	-	-	-	-	1	-
*Weinmannia auriculata* **(T)**	-	-	-	-	-	-	-	-	-	-	-	4	-
*Miconia monzoniensis* **(S)**	-	-	-	-	-	-	-	-	-	-	-	1	-
*Dilleniaceae_tin_1* **(S)**	-	-	-	-	-	-	-	-	-	-	-	+	-
*Clusia trochiformis* **(S)**	-	-	-	-	-	-	-	-	-	-	-	1	-
***Chelyocarpo ulei-Acacietum loretensis* ass. nov.**													
*Myriocarpa stipitata* **(T)**	-	-	-	-	-	-	-	-	-	-	-	-	1
*Clusia amazonica* **(T)**	-	-	-	-	-	-	-	-	-	-	-	-	+
*Heliconia hirsute* **(H)**	-	-	-	-	-	-	-	-	-	-	-	-	1
*Smilax purhampuy* **(C)**	-	-	-	-	-	-	-	-	-	-	-	-	1
*Ochroma pyramidale* **(T)**	-	-	-	-	-	-	-	-	-	-	-	-	2
*Ladenbergia oblongifolia* **(T)**	-	-	-	-	-	-	-	-	-	-	-	-	2
*Urera baccifera* **(S)**	-	-	-	-	-	-	-	-	-	-	-	-	+
*Dichorisandra hexandra* **(C)**	-	-	-	-	-	-	-	-	-	-	-	-	1
*Casearia fasciculata* **(S)**	-	-	-	-	-	-	-	-	-	-	-	-	+
*Boehmeria pavonii* **(S)**	-	-	-	-	-	-	-	-	-	-	-	-	1
*Tectaria antioquoiana* **(H)**	-	-	-	-	-	-	-	-	-	-	-	-	1
*Monstera obliqua* **(H)**	-	-	-	-	-	-	-	-	-	-	-	-	1
*Serpocaulon adnatum* **(H)**	-	-	-	-	-	-	-	-	-	-	-	-	1
*Polybotrya caudata* **(H)**	-	-	-	-	-	-	-	-	-	-	-	-	2
*Chelyocarpus ulei* **(S)**	-	-	-	-	-	-	-	-	-	-	-	-	3
*Selaginella haematodes* **(H)**	-	-	-	-	-	-	-	-	-	-	-	-	2
*Corytoplectus speciosus* **(H)**	-	-	-	-	-	-	-	-	-	-	-	-	1
*Cissus sp._tin_4* **(C)**	-	-	-	-	-	-	-	-	-	-	-	-	+
*Anthurium sp._tin_4* **(H)**	-	-	-	-	-	-	-	-	-	-	-	-	+
*Acacia loretensis* **(T)**	-	-	-	-	-	-	-	-	-	-	-	-	1
*Cecropia aff obtusifolia* **(T)**	-	-	-	-	-	-	-	-	-	-	-	-	+
*Acalypha stricta* **(S)**	-	-	-	-	-	-	-	-	-	-	-	-	1
*Sloanea ptariana* **(T)**	-	-	-	-	-	-	-	-	-	-	-	-	1
*Justicia poeppigiana* **(S)**	-	-	-	-	-	-	-	-	-	-	-	-	+
*Rubiaceae_tin_4* **(S)**	-	-	-	-	-	-	-	-	-	-	-	-	1
*Bauhinia tarapotensis* **(S)**	-	-	-	-	-	-	-	-	-	-	-	-	1
*Perebea angustifolia* **(T)**	-	-	-	-	-	-	-	-	-	-	-	-	1
*Psychotria_tin_4* **(S)**	-	-	-	-	-	-	-	-	-	-	-	-	+
*Paullinia serjaniifolia* **(C)**	-	-	-	-	-	-	-	-	-	-	-	-	1
*Solanum sp._tin_4* **(S)**	-	-	-	-	-	-	-	-	-	-	-	-	+
*Miconia aff abbreviate* **(S)**	-	-	-	-	-	-	-	-	-	-	-	-	1
*Liabum eriocaulon* **(S)**	-	-	-	-	-	-	-	-	-	-	-	-	+
*Gurania rhizantha* **(C)**	-	-	-	-	-	-	-	-	-	-	-	-	+
*Senna multijuga* **(T)**	-	-	-	-	-	-	-	-	-	-	-	-	1
*Inga chartacea* **(T)**	-	-	-	-	-	-	-	-	-	-	-	-	1
*Miconia amplexicaulis* **(S)**	-	-	-	-	-	-	-	-	-	-	-	-	+
*Monnina marginata* **(S)**	-	-	-	-	-	-	-	-	-	-	-	-	+
*Gloxinia xanthophylla* **(H)**	-	-	-	-	-	-	-	-	-	-	-	-	+
*Miconia aff cremophylla* **(S)**	-	-	-	-	-	-	-	-	-	-	-	-	3
*Viburnum hallii* **(S)**	-	-	-	-	-	-	-	-	-	-	-	-	+
*Gunnera sp._tin_4* **(H)**	-	-	-	-	-	-	-	-	-	-	-	-	+
*Gouania aptera* **(S)**	-	-	-	-	-	-	-	-	-	-	-	-	1
*Ficus americana* **(T)**	-	-	-	-	-	-	-	-	-	-	-	-	1
*Gonzalagunia bunchosioides* **(S)**	-	-	-	-	-	-	-	-	-	-	-	-	1

Numbers and symbols are abundance-dominance values of the Braun-Blanquet scale (%): + = <1, 1 = 1 to 5, 2 = 5 to 25, 3 = 25 to 50, 4 = 50 to 75, 5 = >75. The letters in brackets near the species are their life forms: T = Tree, S = Shrub, SS = Subshrub, C = Climber, Ep = Epiphyte, H = Herb. Localities: 2—Ollachea, Camatani, Puno, 13°45′42.77′′ S/70°28′17.06′′ W. 3—San Isidro bridge, San Gabán, Puno, 13°27′58.38′′ S/70°24′56.23′′ W. 4—Payachaca bridge, below Ollachea, Puno, 13°35′06.22′′ S/70°26′26.39′′ W. 5—Ollachea, Puno, 13°46′43.38′′ S/70°28′24.20′′ W. 6—La Coipa, Cajamarca, 5°21′34.88′′ S/78°55′05.08′′ W. 8—San Pedro de la Capilla, Cajamarca, 6°10′04.05′′ S/78°48′49.03′′ W. 9—Gocta, Chachapoyas, Amazonas, 5°57′34.47′′ S/77°51′16.47′′ W. 10—Ocol, Chachapoyas, Amazonas, 6°11′53.27′′ S–77°33′17.47′′ W. 11—Ocol, Chachapoyas, Amazonas, 6°11′24.81′′ S/77°33′30.71′′ W. 12—Satipo, Junín, 11°21′08.60′′ S/74°44′24.32′′ W. 13—Satipo, Junín, 11°14′30.91′′ S/74°40′29.03′′ W. 14—Below Carpish, Huánuco, 9°32′20.31′′ S–76°00′48.02′′ W. 16—Tingo María, Huánuco, 9°10′16.73′′ S–75°53′55.29′′ W.
